# Porcine Invariant Natural Killer T Cells: Functional Profiling and Dynamics in Steady State and Viral Infections

**DOI:** 10.3389/fimmu.2019.01380

**Published:** 2019-06-18

**Authors:** Alexander Schäfer, Jane Hühr, Theresa Schwaiger, Anca Dorhoi, Thomas C. Mettenleiter, Sandra Blome, Charlotte Schröder, Ulrike Blohm

**Affiliations:** ^1^Institute of Immunology, Friedrich-Loeffler-Institut, Greifswald-Insel Riems, Germany; ^2^Department of Experimental Animal Facilities and Biorisk Management, Friedrich-Loeffler-Institut, Greifswald-Insel Riems, Germany; ^3^Institute of Molecular Virology and Cell Biology, Friedrich-Loeffler-Institut, Greifswald-Insel Riems, Germany; ^4^Institute of Diagnostic Virology, Friedrich-Loeffler-Institut, Greifswald-Insel Riems, Germany

**Keywords:** iNKT cells, T cells, pig, biomedical model, influenza A virus, African swine fever virus

## Abstract

Pigs are important livestock and comprehensive understanding of their immune responses in infections is critical to improve vaccines and therapies. Moreover, similarities between human and swine physiology suggest that pigs are a superior animal model for immunological studies. However, paucity of experimental tools for a systematic analysis of the immune responses in pigs represent a major disadvantage. To evaluate the pig as a biomedical model and additionally expand the knowledge of rare immune cell populations in swine, we established a multicolor flow cytometry analysis platform of surface marker expression and cellular responses for porcine invariant Natural Killer T cells (iNKT). In humans, iNKT cells are among the first line defenders in various tissues, respond to CD1d-restricted antigens and become rapidly activated. Naïve porcine iNKT cells were CD3^+^/CD4^−^/CD8^+^ or CD3^+^/CD4^−^/CD8^−^ and displayed an effector- or memory-like phenotype (CD25^+^/ICOS^+^/CD5^hi^/CD45RA^−^/CCR7 ^±^ /CD27^+^). Based on their expression of the transcription factors T bet and the iNKT cell-specific promyelocytic leukemia zinc finger protein (PLZF), porcine iNKT cells were differentiated into functional subsets. Analogous to human iNKT cells, *in vitro* stimulation of porcine leukocytes with the CD1d ligand α-galactosylceramide resulted in rapid iNKT cell proliferation, evidenced by an increase in frequency and Ki-67 expression. Moreover, this approach revealed CD25, CD5, ICOS, and the major histocompatibility complex class II (MHC II) as activation markers on porcine iNKT cells. Activated iNKT cells also expressed interferon-γ, upregulated perforin expression, and displayed degranulation. In steady state, iNKT cell frequency was highest in newborn piglets and decreased with age. Upon infection with two viruses of high relevance to swine and humans, iNKT cells expanded. Animals infected with African swine fever virus displayed an increase of iNKT cell frequency in peripheral blood, regional lymph nodes, and lungs. During Influenza A virus infection, iNKT cell percentage increased in blood, lung lymph nodes, and broncho-alveolar lavage. Our in-depth characterization of porcine iNKT cells contributes to a better understanding of porcine immune responses, thereby facilitating the design of innovative interventions against infectious diseases. Moreover, we provide new evidence that endorses the suitability of the pig as a biomedical model for iNKT cell research.

## Introduction

Biomedical research is in need of large animal models that reflect human infectious diseases better than current rodent models (1, 2). Because of the striking resemblances between porcine and human immune system and physiology, pigs could be a superior model species (3–10). In order to establish pigs as a new biomedical model species, a more detailed understanding of porcine immune responses and leukocyte subsets is strongly needed.

Leukocytes at systemic and peripheral sites are eminently important for control of microbial colonization and defense against infections by induction of protective immunity (11). One of those leukocyte subsets are invariant Natural Killer T (iNKT) cells. These cells bridge and orchestrate both untargeted innate and specific adaptive responses, which are crucial for pathogen clearance and survival. In contrast to the vast heterogeneity of T cell receptors (TCR) among conventional CD3^+^ T cells (cTC), iNKT cells possess a semi-invariant TCR. This TCR is restricted to the non-classical major histocompatibility complex (MHC) class I-related CD1d, presenting lipid or glycolipid antigens. iNKT cells can be activated antigen-dependently with glycolipids derived from microbes or the host by TCR-CD1d interactions or antigen-independently via cytokines, mainly interleukin-(IL-)12 and IL-18 or type I interferons (IFN). The induction of cytokine expression in iNKT cells does not require classical co-stimulation, whereas iNKT cell proliferation depends on co-stimulatory signals by B7/CD28 or CD40/CD40L (12). Additionally, effector cytokines are present as immediately available preformed mRNA transcripts in iNKT cells (13). Therefore, iNKT cells rapidly proliferate and secrete effector molecules like IFNγ, IL-17 or granulocyte-macrophage colony-stimulating factor after activation (14). Moreover, they are able to lyse infected cells by perforin and Fas/FasL interaction (15–18). iNKT cells also augment B cell responses, class switching and affinity maturation independently of classical helper cells (19, 20). Several iNKT cell subsets have been identified by their expression of different transcription factors, like promyelocytic leukemia zinc finger protein (PLZF) and T-bet, most notably in mice, where they are primarily differentiated into iNKT1 (T-bet^+^/PLZF^+^), iNKT2 (T-bet^−^/PLZF^hi^), and iNKT17 (T-bet^−^/PLZF^lo^) (21–26). Differentiated subsets of human iNKT cells are not as well-defined as in mice (27).

At present, most studies focusing on porcine iNKT cells investigated the potential use of the cognate CD1d ligand α-galactosyl-C16-ceramide (αGC) as an adjuvant for vaccines against Influenza A virus (IAV) and other infectious diseases (28–32). Knowledge about phenotype, response kinetics, and functional aspects of iNKT cell responses in pigs is scarce. The antigen presentation molecules of the CD1 family are of particular importance for iNKT cells. In addition to CD1d, there are four other members in the CD1 family, CD1a, CD1b, CD1c, and CD1e. All of these molecules are expressed in pigs as well as in humans, while mice lack proteins other than CD1d (33). It has been shown that expression of CD1d on thymocytes is required for iNKT cell development, in mice (34) as well as in pigs (35). Murine CD1d tetramers loaded with the αGC analog PBS57 have been shown to detect porcine iNKT cells (7, 36). Their frequency is typically between 0.01 and 1% among CD3^+^ T cells, thereby resembling human iNKT cell frequencies (7, 28). Porcine iNKT cells are CD8α^+^ or CD8α^−^ but lack expression of CD4 in most tissues (28, 29, 37). In contrast to human and murine iNKT cells, naïve porcine iNKT cells express high levels of CD44, as most lymphocytes in swine (38, 39). Moreover, the iNKT cell-specific transcription factor PLZF is highly expressed in porcine iNKT cells (36). Some evidence indicates that iNKT cells expand upon stimulation of porcine PBMC with αGC (29, 36). Using next generation sequencing, it has recently been shown that porcine iNKT cells predominantly use Vα and Jα segments homologous to the Vα24-Jα18 and Vα14-Jα18 rearrangements used in humans and mice, respectively (40). Moreover, molecular investigations demonstrated that the antigen-binding domain of the invariant α-chain, CDR1α, is conserved between pigs and humans (40). This indicates that responses of porcine iNKT cells mimic responses of human iNKT cells, thereby further underlining the suitability of the pig as a biomedical model species for human iNKT cells.

In order to comprehensively understand porcine iNKT cells and advance research, we investigated their phenotype, dynamics and functional responses in-depth in steady state and during IAV and African swine fever virus (ASFV) infection.

## Materials and Methods

### Pigs and Biological Samples

In total 13 German landrace pigs for IAV and 12 for ASFV experiments were obtained from a commercial breeding unit (BHZP-Basiszuchtbetrieb Garlitz-Langenheide, Germany) with high biosecurity standards and hygiene (free of IAV and Porcine reproductive and respiratory syndrome virus among others). Samples for the investigation of age-dependent changes in iNKT cell frequency were obtained during routine veterinary check-ups from the same commercial breeding unit (BHZP-Basiszuchtbetrieb Garlitz-Langenheide, Germany). Swine (Danish landrace/Danish Large White/Danish Duroc hybrid) used for *in vitro* experiments involving PBMC, were kept at the Friedrich-Loeffler-Institut (FLI), Greifswald-Insel Riems under conventional conditions.

### Viruses and Infection Experiments

Influenza virus A/Bayern/74/2009 was propagated on Madin-Darby canine kidney cells (MDCKII) cells in MEM supplemented with 0.56% bovine serum albumin, 100 U/ml Penicillin, 100 μg/ml Streptomycin and 2 μg/ml L-1-Tosylamide-2-phenylethyl chloromethyl ketone (TPCK)-treated trypsin (Sigma-Aldrich, USA). For viral titration by TCID_50_ assay, serial 10-fold dilutions of virus suspensions were prepared, added to MDCKII cells in 96-well plates, and incubated for 3 days at 37°C and 5% CO_2_. Cytopathic effect was microscopically evaluated. Titers were calculated according to Spearman-Kärber (41, 42). Four-week-old piglets were obtained from a commercial breeding facility directly after weaning. Absence of acute IAV infection of pigs used for the IAV study was confirmed by real-time PCR (AgPath.IDTM One-Step RT-PCR Kit, Applied Biosystems, USA) of nasal swabs prior to transport to the FLI (modified from Spackman et al. (43)). IAV infection was performed 3 weeks after transport to our facility by intranasal administration of 2 ml virus suspension (10^6^ TCID_50_/ml) using mucosal atomization devices (Wolfe Tory Medical, USA).

ASFV Armenia08 was propagated and titrated using mature porcine PBMC-derived macrophages as previously described (44). For back titration, virus hemadsorption test was performed by endpoint titration of the diluted inoculation virus. In brief, 100 μl virus dilution were incubated for 24 h on PBMC-derived macrophages in 96-well plates. Thereafter, 20 μl of a 1% homolog erythrocyte suspension were added and hemadsorption read after 24 and 48 h. All samples were tested in quadruplicates. Hemadsorbing units (HAU) were used for read-out. For infection, 2 ml macrophage culture supernatant containing 10^6.25^ HAU ASFV Armenia08 were inoculated oro-nasally. All work involving ASFV was done in the high containment facility (L3^+^) at the Friedrich-Loeffler-Institut.

### Cell Isolation and Culture

For isolation of peripheral blood mononuclear cells (PBMC), whole blood was separated by density gradient centrifugation using Pancoll (PAN-Biotech, Germany). PBMC were collected and washed with PBS-EDTA (1 mM; used for all analyses). Cell count was determined using Neubauer improved haemocytometer. Single cell suspensions from spleen and lymph nodes were prepared by mechanically disrupting tissue with a sieve. Lymphocytes from liver were isolated following a modified protocol previously described (45). In brief, liver samples were perfused with ice-cold PBS-EDTA. Perfused regions were minced with sterile scissors, resuspended in PBS-EDTA supplemented with 100 μM CaCl_2_, and digested with Collagenase D (1 mg/ml; Sigma-Aldrich) for 40 min at 37°C. Remaining tissue was removed by short centrifugation. Cell pellet was resuspended in PBS-EDTA and used for flow cytometry. Lymphocytes from lung tissue were isolated by mincing non-perfused lung tissue, followed by enzymatic digestion as described for liver samples. Lung tissue was additionally mashed through a cell strainer with the plunger of a syringe after digestion. Unless otherwise stated, cells were cultured in Ham's F12/IMDM (1:1), supplemented with 10% fetal calf serum (FCS), 2-mercaptoethanol (50 μM), 100 U/ml penicillin, and 100 μg/ml streptomycin.

### Cell Stimulation

For iNKT cell stimulation, freshly isolated PBMC were seed into round-bottom 96-well plates at a density of 10^7^ PBMC/ml. αGC (Toronto Research Chemicals, Canada) dissolved in dimethyl sulfoxide (DMSO; Sigma-Aldrich, USA) or DMSO as vehicle control diluted in cell culture media were added. αGC was used at 0.1 μg/ml (low-dose) and 1 μg/ml (high-dose). Cells were incubated at 38.5°C, 5% CO_2_ for the indicated time. After incubation, cells were harvested, washed with PBS-EDTA, and analyzed by flow cytometry.

For detection of IFNγ and perforin, cells were stimulated as previously described (46). After 4 days, fresh medium with αGC in the corresponding concentrations was added to the cells. After another 2 h incubation, Brefeldin A (10 μg/ml, Biolegend, USA) was added to enable intracellular accumulation of target proteins. Cells were incubated for 4 h and then stained and analyzed by flow cytometry.

For analysis of CD107a surface expression as a marker of degranulation, cells were treated as previously described (47). Briefly, freshly isolated PBMC were seed into 96-well plates at a density of 10^7^ PBMC/ml and rested overnight. For antigenic stimulation, αGC was added for a final concentration of 0.1 μg/ml or 1 μg/ml. As unspecific inducers, Phorbol-12-myristat-13-acetat (PMA; Sigma-Aldrich, USA) and ionomycin (Sigma-Aldrich, USA) were added for final concentrations of 50 ng/ml and 1 μg/ml, respectively. The cells were stimulated in the presence of anti-CD107a antibodies (clone 4E9/11, Bio-Rad, USA; 4 μg/ml). After 1 h incubation, Brefeldin A (10 μg/ml), and Monensin (4 μM, Biolegend, USA) were added and the cells were incubated for another 5 h and then stained and analyzed by flow cytometry. Specific degranulation was calculated as the difference in surface expression of stimulated and control cells and is given as ΔCD107a.

For *in vitro* activation assays, porcine CD172a^+^ cells were purified using monoclonal antibodies (cone 74-22-15) and magnetic anti-mouse IgG1 beads (BD Bioscience, USA). CD172a^+^ cells were infected with IAV (MOI 1) or ASFV (MOI 0.1) for 48 h. Then, supernatants were collected, cleared of debris by centrifugation, and stored at −80°C until further use. Freshly isolated porcine PBMC were stimulated with the respective supernatants or 1 μg/ml αGC as a positive control for 4 days and then stained and analyzed by flow cytometry.

### Cell Proliferation Assay

Freshly isolated PBMC were stained using Tag-it Violet Proliferation and Cell Tracking Dye (Biolegend, USA) according to the manufacturer's instructions. Briefly, total PBMC were adjusted to 1 × 10^7^ cells/ml in PBS and incubated with the Tag-it Violet Proliferation and Cell Tracking Dye at a concentration of 5 μM. Cells were incubated for 20 min at 37°C in the dark. Quenching was done by addition of cell culture media supplemented with 10% FCS. After washing, stained cells were used for visualization of iNKT cell proliferation after stimulation with αGC.

### Flow Cytometry

Stainings for flow cytometry were performed using either single cell suspensions or whole blood. All incubation steps were carried out for 15 min at 4°C in the dark, unless otherwise stated. Antibodies used for flow cytometry are shown in Supplementary Table 1. For iNKT cell staining, first tetramers were added at the predetermined concentration (1:500) and incubated at room temperature in the dark for 30 min. Antibodies for staining of additional surface markers were added without washing and incubated with the tetramers for another 15 min. After washing, antibody staining was proceeded. Unconjugated antibodies were detected by isotype-specific fluorochrome-conjugated secondary antibodies. When whole blood or tissue samples containing erythrocytes were stained, erythrocytes were lysed after surface staining prior to fixation by lysis buffer (1.55 M NH_4_Cl, 100 mM KHCO_3_, 12.7 mM Na_4_EDTA, pH 7.4, in A.dest.). For intracellular staining, cells were fixed after surface staining using the True-Nuclear Transcription Factor Buffer Set (Biolegend, USA) according to the manufacturer's instructions. Murine CD1d tetramers, empty or loaded with PBS57, were obtained from the NIH Tetramer Core Facility.

Doublets were excluded by consecutive gating FSC-W/FSC-H and SSC-W/SSC-H. Living lymphocytes were gated based on their forward-scatter (FSC) and side-scatter (SSC) properties. Live, single lymphocytes were further separated in conventional T cells (cTC; CD3^+^/CD1d-Tet^−^) and iNKT cells (CD3^+^/CD1d-Tet^+^) for subsequent analysis.

Per sample, at least 1 × 10^5^ single cTC were recorded. BD FACS Canto II or BD LSRFortessa with FACS DIVA Software (all BD Bioscience, USA) and FlowJo V10 (Treestar, USA) were used for all analyses.

### Statistical Analysis

GraphPad Prism 7 (Graphpad Software Inc., USA) was used for statistical analysis and graph creation. Normality was tested with the Shapiro-Wilk normality test. Subsequent analysis was performed with either parametric tests for normally distributed data sets or non-parametric tests for non-normally distributed data sets. For analysis of data sets with three or more groups, Repeated-measures one-way ANOVA was used to investigate statistically significant differences between the groups. Multiple comparisons were performed for differences between iNKT cells and cTC subsets, respectively. No statistical analyses were performed for differences between iNKT cells and cTC. Holm-Sidak's *post-hoc* test was used for correction of multiple comparisons. For analysis of data from the IAV and ASFV trial, ordinary one-way ANOVA with Holm-Sidak's *post-hoc* test for correction of multiple comparisons was used. For analysis of data sets of two groups, paired *t*-tests were used. Unless indicated otherwise, data is shown as mean (SD). Statistical significance was defined as ^*^p < 0.05, ^**^p < 0.01, ^***^p < 0.001, and ^****^p < 0.0001.

## Results

### Naïve Peripheral Porcine iNKT Cells Are Mostly CD8α^+^ and Display an Effector- and Memory-Like Phenotype

**Figure 1:**
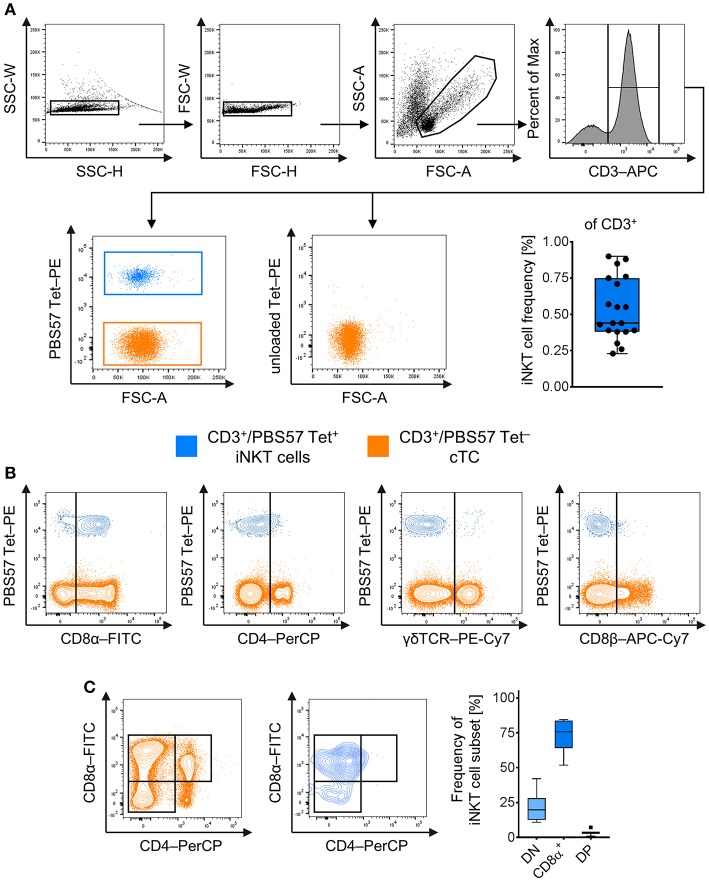
Identification and phenotype of naïve peripheral porcine iNKT cells. Expression of surface markers was analyzed by flow cytometry. **(A)** Doublets were excluded by SSC-W vs. SSC-H gates, followed by FSC-W vs. FSC-H gates. Live lymphocytes were identified according to their FSC/SSC characteristics. All T cells were identified using antibodies against CD3. CD3^+^ T cells stained with the PBS57-loaded CD1d tetramer (left plot) were defined as invariant Natural Killer T cells (iNKT; blue), tetramer-negative cells were defined as conventional T cells (cTC; orange). Unloaded tetramers served as control (right plot). Frequency of iNKT cells in peripheral blood among CD3^+^ lymphocytes shown as Tukey box plot (*n* = 19). **(B)** Evaluation of expression of CD8α, CD4, γδTCR, and CD8β on iNKT cells and cTC. Representative plots of at least four experiments are shown. **(C)** Representative plots of CD8α and CD4 co-expression by cTC and iNKT cells. CD8α^−^/CD4^−^ (DN; light blue), CD8α^+^/CD4^−^ (CD8α^+^, blue), and CD8α^+^/CD4^+^ (DP; dark blue) subsets among iNKT cells were identified according to their expression pattern in cTC. Frequency of iNKT cell subsets shown as Tukey box plots (*n* = 7).

To investigate porcine iNKT cells in peripheral blood by flow cytometry, we used murine CD1d tetramers loaded with the αGC analog PBS57 (PBS57 Tet) as previously described (7, 36). After exclusion of doublet cells, live cTC were gated as CD3^+^/PBS57 Tet^−^ cells and iNKT cells were defined as CD3^+^/PBS57 Tet^+^ among the lymphocyte gate [Figure 1A (48)]. In healthy animals, the average iNKT cell frequency was 0.5%, comparable to frequencies in blood published earlier (36, 37). Most naïve iNKT cells expressed CD8α, while only a small amount was CD4^+^, although a considerable proportion of iNKT cells expressed low levels of CD4 (Figures 1B,C). Thereby, we confirmed previous findings showing similar results (36, 37). iNKT cells expressed neither CD8β nor γδTCR (Figure 1B). For differentiation of iNKT cell subsets, expression of the classical T cell markers CD8α and CD4 was analyzed. The subsets were gated according to the respective expression of these markers on cTC. The major subset of peripheral iNKT cells in swine was CD8α^+^/CD4^−^ (73.7 ± 11.9%; Figure 1C). There was no distinct CD4^+^ population; however, a minor fraction of iNKT cells was CD8α^+^/CD4^lo^ (DP; 3.6 ± 1.9%). Moreover, there was a considerable CD8^−^/CD4^−^ (DN) population (21.5 ± 10.8%).

**Figure 2:**
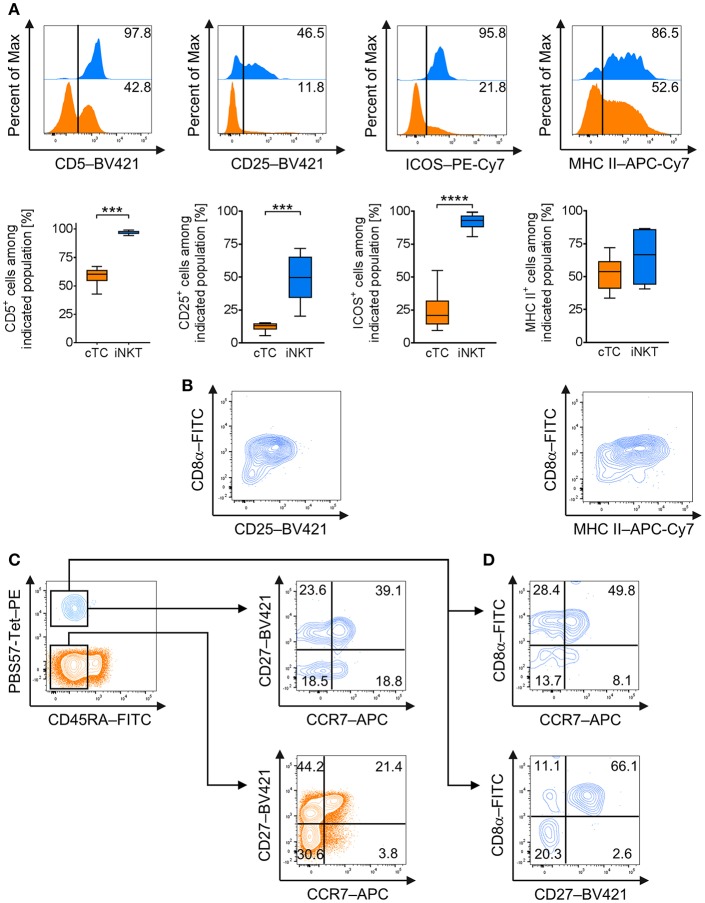
Expression of effector and memory cell-associated markers on naïve porcine iNKT cells. Expression of surface markers associated with effector functions and memory status on naïve peripheral iNKT cells was analyzed by flow cytometry. **(A)** Representative flow cytometric plots of CD5, CD25, MHC II, and ICOS expression by iNKT cells (blue) and cTC (orange). Summarized data show frequencies of iNKT cells or cTC expressing CD5, CD25, MHC II, and ICOS (mean (SD), *n* = 6). **(B)** Representative flow cytometric plots showing differential expression of CD8a and CD25 or MHC II on iNKT cells. **(C)** Expression of CD45RA (left) on iNKT cells and cTC. CD45RA^−^ cTC (middle) and iNKT cells (right) were further investigated for expression of CD27 and CCR7 and divided into CD27^+^/CCR7^+^ central memory cells, CD27^+^/CCR7^−^ transitional memory cells, CD27^−^/CCR7^−^ effector memory cells, and CD27^−^/CCR7^+^ activated effector memory cells. **(D)** Differential expression of CD8α and CCR7 or CD27. Representative plots and histograms of at least three experiments are shown. ^***^p < 0.001, ^****^p < 0.0001, paired *t*-test.

For further differentiation, we investigated steady state expression of surface markers that are frequently associated with an effector phenotype in mice and humans, i.e., CD5 (49), CD25 [IL-2Rα (50)], CD278 [Inducible T-cell co-stimulator, ICOS (51)], and the major histocompatibility complex class II [MHC II (52)] in naïve iNKT cells and cTC. All iNKT cells were positive for CD5 whereas only around 60% of cTC showed CD5 expression (Figure 2A). Around half of all iNKT cells expressed CD25 at low levels on their surface, while cTC displayed only a minor CD25^hi^ fraction (Figure 2A). ICOS was expressed on virtually all iNKT cells, while only on a minor fraction of cTC (Figure 2A). MHC II was expressed on most iNKT cells at medium or high levels (Figure 2A). In contrast, cTC were mostly MHC II^−^ but the MHC II^+^ fraction expressed it at comparable levels (Figure 2A). Because CD25 and MHC II were not expressed by all iNKT cells, we investigated which iNKT cell subset expressed the proteins (Figure 2B). Differential staining with CD8α revealed that both proteins were predominantly expressed on CD8α^+^ iNKT cells. DN iNKT cells showed no or low expression of CD25 and MHC II.

To investigate the functional differentiation of porcine iNKT cells, we analyzed the expression of markers regularly associated with antigen experience and memory status, CD45RA, C-C chemokine receptor type 7 (CCR7), and CD27 (53–56). cTC displayed a minor fraction of naïve CD45RA^+^ cells, while the larger fraction consisted of antigen-experienced CD45RA^−^ cells (Figure 2C). CD45RA^−^ cTC were further divided into CD27^+^/CCR7^+^ central memory cells, CD27^+^/CCR7^−^ transitional memory cells and CD27^−^/CCR7^−^ effector memory cells. There was only a minor population of CD27^−^/CCR7^+^ activated effector memory cells (Figure 2C). All iNKT cells were CD45RA^−^ and thus displayed an antigen-experienced phenotype. Most iNKT cells were CD27^+^/CCR7^+^, resembling central memory cells. A second major fraction expressed CD27 but was CCR7^−^, similar to transitional memory cells. About a third of iNKT cells was CD27^−^ and could further be divided into equal fractions of CCR7^−^ and CCR7^+^, thereby differentiating subsets comparable to resting and activated effector memory cells, respectively (Figure 2C). Expression of CD27 and CCR7 was also investigated for co-expression with CD8α^+^. Most CD8α^+^ iNKT cells expressed CCR7 as well as CD27 (Figure 2D). DN iNKT cells were negative for CD27 while a fraction expressed CCR7 (Figure 2D).

**Figure 3:**
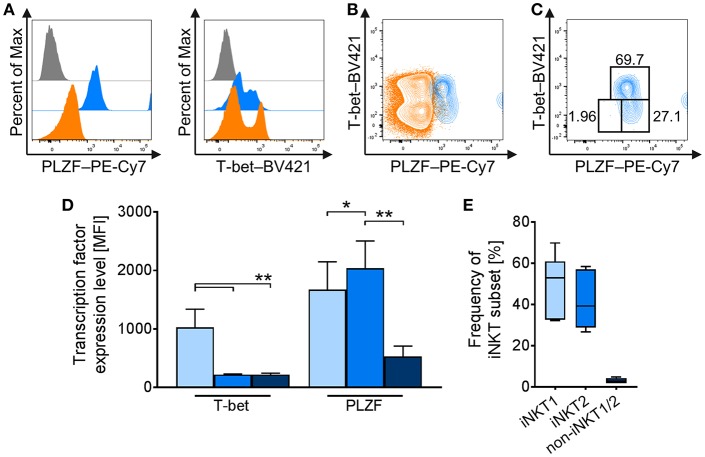
Differential profiling of naïve porcine iNKT cells. Peripheral iNKT cells were differentiated by flow cytometry according to their expression of the transcription factors PLZF and T-bet. **(A)** Representative flow cytometric histograms of PLZF (left) and T-bet (right) expression in iNKT cells (blue) and cTC (orange). Control stainings are shown in gray. **(B)** Representative flow cytometric plot showing co-expression of T-bet and PLZF in iNKT cells and cTC. **(C)** Differential gating of iNKT cells according to their T-bet and PLZF expression. iNKT1 were defined as T-bet^+^/PLZF^+^ (light blue), iNKT2 as T-bet^−^/PLZF^hi^ (blue), and non-iNKT1/2 as T-bet^−^/PLZF^lo^ (dark blue). **(D)** Expression profiles of T-bet and PLZF in iNKT1 (light blue), iNKT2 (blue), and non-iNKT1/2 (dark blue) (*n* = 6). **(E)** Frequency of iNKT subsets in naïve swine shown as Tukey box plots (*n* = 6). ^*^p < 0.05, ^**^p < 0.01, repeated-measures one-way ANOVA with Holm-Sidak's *post-hoc* test for multiple comparisons.

In mice, intracellular staining of transcription factors, including T-bet and PLZF, is used to define functional iNKT cell subsets iNKT1 (T-bet^+^/PLZF^+^), iNKT2 (T-bet^−^/PLZF^hi^), and iNKT17 (T-bet^−^/PLZF^lo^) resembling the T-helper cell populations Th1, Th2, and Th17, respectively (21–26). Currently, there is no such differentiation available for porcine iNKT cells. Therefore, we investigated the expression of T-bet and PLZF in porcine iNKT cells. We confirmed that porcine iNKT cells express higher levels of PLZF than cTC (Figure 3A). Moreover, we detected expression of T-bet in a subset of porcine iNKT cells (Figure 3A). Co-expression of T-bet and PLZF was used to define iNKT cell subsets corresponding to the ones described in other species (Figures 3B–E). In naïve swine, the largest iNKT cell subset had a T-bet^+^/PLZF^+^ phenotype and was therefore defined as iNKT1 (49.8 ± 14.8%). The second major group was T-bet^−^/PLZF^hi^, thereby resembling iNKT2 (41.7 ± 13.3%). A small fraction was T-bet^−^/PLZF^lo^ (3.1 ± 1.2%) and was defined as non-iNKT1/non-iNKT2 (non-iNKT1/2) because additional differentiating markers were not available. Appropriate differentiation was confirmed by analysis of T-bet and PLZF expression levels in the iNKT cell subsets. T-bet expression in iNKT1 was significantly higher than in iNKT2 and non-iNKT1/2 (Figure 3D). PLZF expression level was the highest in iNKT2 and the lowest in non-iNKT1/2 (Figure 3D).

Taken together, we confirmed the phenotype of naïve iNKT cells from peripheral blood of healthy pigs to be predominantly CD3^+^/CD4^−^/CD8^+^ or CD3^+^/DN. All iNKT cells expressed the αβTCR but not the CD8 β-chain. Moreover, we showed that naïve porcine iNKT cells display an effector-like (CD5^hi^/CD25^+^/MHC II^+^/ICOS^+^) and memory-like phenotype (CD45RA^−^/CCR7^+^/CD27^+^). The CD8α subset expressed higher levels of CD25 and MHC II, as well as higher levels of CCR7 and CD27. Additionally, analogous to classifications in rodents, we were able to divide peripheral iNKT cells into three subsets, iNKT1, iNKT2, and non-iNKT1/2, according to their respective T-bet and PLZF expression.

### Antigenic Activation Induces Strong Proliferation in Porcine iNKT Cells

**Figure 4:**
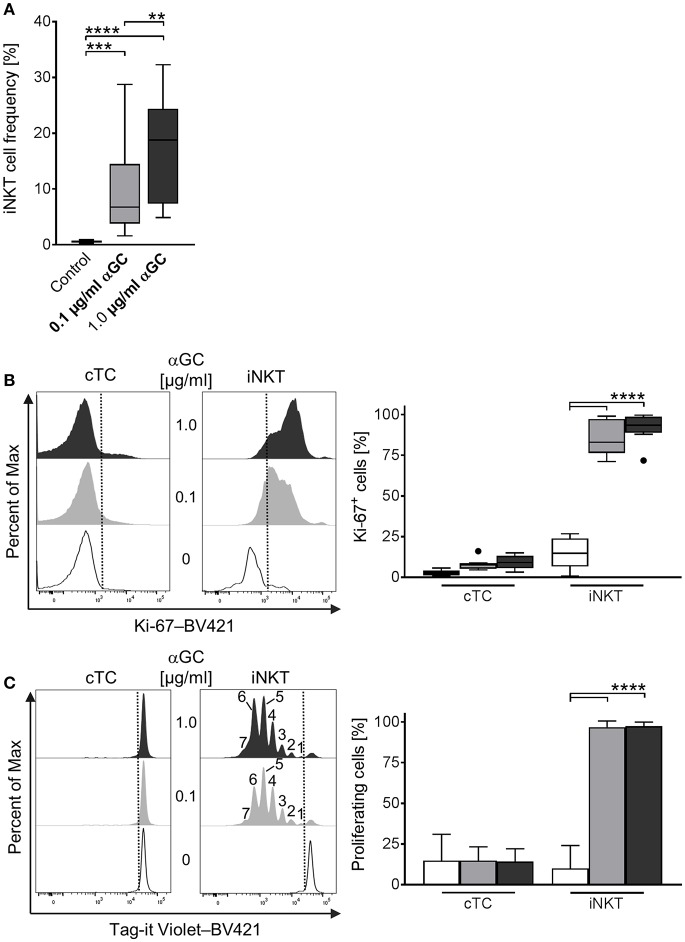
Proliferative activity of porcine iNKT cells upon antigenic activation. **(A)** PBMC were cultivated in the presence of 0.1 μg/ml (gray) or 1 μg/ml (black) αGC or DMSO as vehicle control (white) for 4 days. Proliferation measured as iNKT cell frequency among CD3^+^ lymphocytes shown as Tukey box plots (*n* = 17). **(B)** Cell proliferation was investigated by measuring Ki-67 expression in cTC and iNKT cells. Representative histograms showing expression of Ki-67 in cTC (left) and iNKT cells (right). Dotted lines indicate the threshold according to the control staining. Frequency of proliferating, Ki-67^+^ iNKT cells and cTC (*n* = 6). **(C)** Porcine PBMC were stained with Tag-it Violet and cultivated in the presence of 0.1 μg/ml (gray) or 1 μg/ml (black) αGC or DMSO as vehicle control (white) for 4 days. Proliferating cells were defined as Tag-it Violet^lo^. Representative histograms showing proliferating, Tag-it Violet^lo^ cells in cTC and iNKT cells. Dotted lines indicate the threshold according to the control staining. Numbers indicate individual proliferation steps. Frequency of proliferating, Tag-it Violet^lo^ cTC and iNKT cells (mean (SD), *n* = 3). ^**^p < 0.01, ^***^p < 0.001, ^****^p < 0.0001, repeated-measures one-way ANOVA with Holm-Sidak's *post-hoc* test for multiple comparisons.

A major feature of iNKT cells is their ability to proliferate rapidly upon activation. For porcine iNKT cells, data regarding responses after antigenic activation is limited. Therefore, we investigated the proliferative ability by quantifying iNKT frequency among CD3^+^ lymphocytes and expression of the proliferation marker Ki-67 after stimulation of porcine PBMC with low-dose (0.1 μg/ml) and high-dose (1 μg/ml) αGC or DMSO as vehicle control for 4 days. We also visualized cell divisions by staining with a fluorescent cell tracking dye. Upon stimulation with αGC, iNKT cell frequency among CD3^+^ lymphocytes strikingly increased. After 4 days of stimulation with low-dose αGC, iNKT cells accounted for about 9.7 ± 7.9% of CD3^+^ T cells, a 20-fold increase. High-dose αGC stimulation resulted in a 30-fold increase, leading to an iNKT cell frequency of 16.9 ± 9% among CD3^+^ lymphocytes (Figure 4A). To verify that this increase was due to proliferation, we investigated the expression of Ki-67, a widely used marker specific for proliferating cells (57). Most iNKT cells were Ki-67^+^ after stimulation (85.3 ± 10.6% and 91.7 ± 8.7% after low- and high-dose αGC stimulation, respectively), thereby demonstrating that the majority of iNKT cells were proliferating (Figure 4B). Expression of Ki-67 in cTC remained on a low level of background activation, proving the specific activation of iNKT cells by αGC (Figure 4B). Staining of PBMC with a fluorescent cell tracking dye (Tag-it Violet) before stimulation allows for detection of single proliferation steps upon activation. Again, nearly all iNKT cells were in a highly proliferative state and up to seven proliferation steps were detectable (Figure 4C). There was no detectable difference between both αGC doses. cTC demonstrated no proliferation over background level upon αGC stimulation.

Taken together, we demonstrated the rapid proliferative abilities of peripheral porcine iNKT cells in response to antigenic stimulation.

### Porcine iNKT Cells Upregulate Expression of CD25, MHC II, ICOS, and CD5 and Differentiate Into iNKT1 Upon Antigenic Stimulation

**Figure 5:**
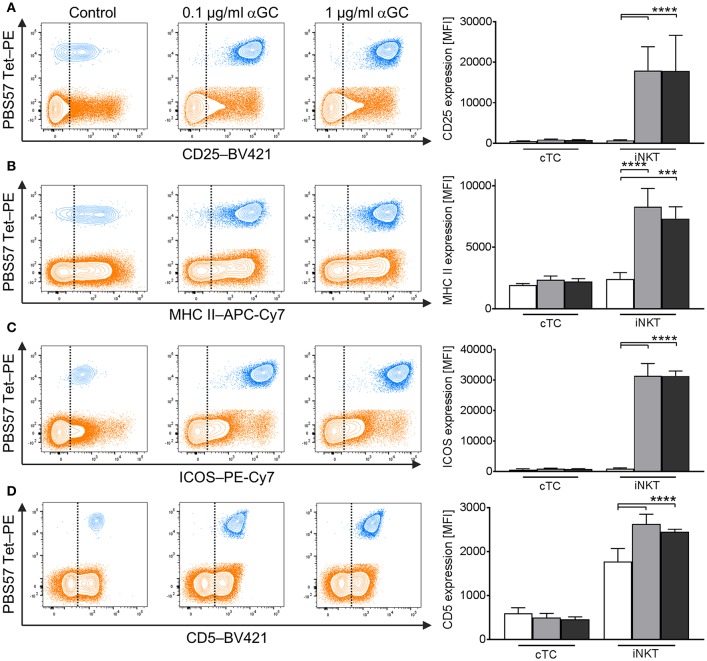
Changes in surface marker expression on porcine iNKT cells upon antigenic activation. Porcine PBMC were incubated in the presence of 0.1 μg/ml or 1 μg/ml αGC or DMSO as vehicle control for 4 days. Representative flow cytometric plots showing expression of **(A)** CD25, **(B)** MHC II, **(C)** ICOS, and **(D)** CD5 on cTC (orange) and iNKT cells (blue). Expression level of control cells (white) and cells stimulated with 0.1 μg/ml (gray) and 1 μg/ml αGC (black) are shown (*n* = 3-4). Dotted lines show threshold according to marker expression by cTC. Data shown as mean (SD), ^***^p < 0.001, ^****^p < 0.0001, repeated-measures one-way ANOVA with Holm-Sidak's *post-hoc* test for multiple comparisons.

Whereas several iNKT cell activation markers are known in mice and humans, for porcine iNKT cells, no markers have been described to investigate their immune response or differentiation status. We therefore analyzed low- and high-dose αGC-stimulated porcine PBMC for activation-dependent changes in expression of multiple surface and intracellular markers. Among the established activation markers used for human and murine T cells are CD25 (50), MHC II (52), ICOS (51), and CD5 (49). Multicolor flow cytometry revealed that the expression of these markers on the surface of cTC did not change significantly irrespective of stimulus or dose. In contrast, upon activation, iNKT cells significantly upregulated the expression of all investigated surface markers. Since most naïve iNKT cells were already positive for MHC II, ICOS, and CD5, the percentage of cells positive for the respective markers did not change. Only the frequency of CD25^+^ iNKT cells increased significantly after stimulation. However, the expression level of all markers on the surface of iNKT cells increased significantly upon αGC stimulation, as evidenced by heightened MFI. CD25 expression (Figure 5A) increased from controls (MFI: 591 ± 106) to low-dose and high-dose αGC-stimulated iNKT cells (MFI: 15,427 ± 3,717; 13,593 ± 2,568, respectively). MHC II (Figure 5B) was markedly upregulated between controls (MFI: 2,341 ± 1,248) to low-dose (MFI: 9,653 ± 1,527) and high-dose αGC-stimulated iNKT cells (MFI: 8,159 ± 1,215). ICOS expression (Figure 5C) rose from controls (MFI: 849 ± 678) to low-dose (MFI: 35,309 ± 2,548) and high-dose αGC-stimulated iNKT cells (MFI: 31,366 ± 4,013). Expression levels of CD5 (Figure 5D) increased from controls (MFI: 1,776 ± 294) to low-dose (MFI: 2,626 ± 170) and high-dose αGC-stimulated iNKT cells (MFI: 2,448 ± 59). There were no significant differences in the expression levels of CD25, MHC II, ICOS, and CD5 on cTC after stimulation with low or high concentrations of αGC.

**Figure 6:**
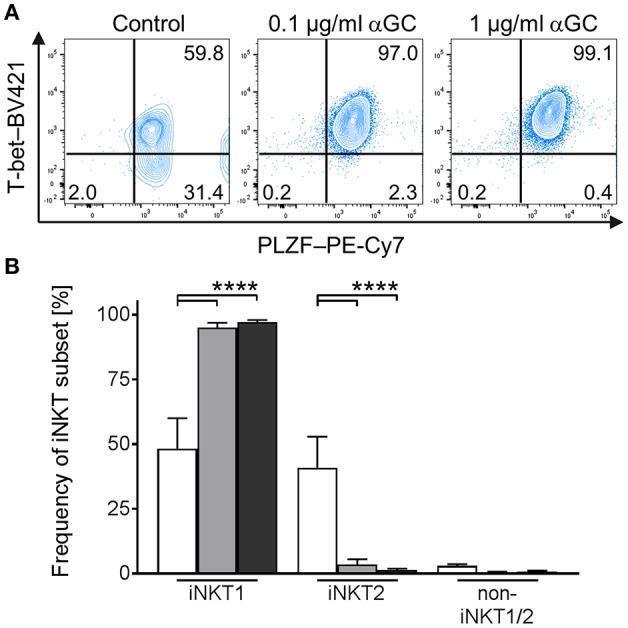
Differentiation of activated porcine iNKT cells. Porcine PBMC were cultivated in the presence of αGC or DMSO as vehicle control. Intracellular expression of T-bet and PLZF was analyzed by flow cytometry after stimulation for 4 days. **(A)** Representative plots of T-bet/PLZF co-expression in untreated control iNKT cells (left) and iNKT cells stimulated with 0.1 μg/ml (middle) or 1 μg/ml (right) αGC. **(B)** Frequency of iNKT1, iNKT2, and non-iNKT1/2 upon stimulation with 0.1 μg/ml (gray) or 1 μg/ml (black) αGC or DMSO as vehicle control (white) (*n* = 6). Data shown as mean (SD), ^****^p < 0.0001, repeated-measures one-way ANOVA with Holm-Sidak's *post-hoc* test for multiple comparisons.

In mice, activated iNKT cells have been shown to regulate the expression of T-bet and PLZF, depending on the type of activating stimulus (21–26). Comparable data for swine is missing. To investigate the differentiation status of αGC-activated porcine iNKT cells, we used the differential staining of T-bet and PLZF established in this study. While PLZF expression was not regulated in iNKT cells (Figure 6A), T-bet expression increased in iNKT cells after stimulation with low- and high-dose αGC (Figure 6A). Co-expression analysis revealed that all αGC-activated iNKT cells were T-bet^+^/PLZF^+^ iNKT1 (Figure 6B). There was no difference between low- and high-dose stimulated iNKT cells.

In short, we established a stimulation protocol for porcine iNKT cells. Activation by αGC results in strong upregulation of CD5, CD25, ICOS, and MHC II on iNKT cells and differentiation into T-bet^+^/PLZF^+^ iNKT1.

### Porcine iNKT Cells Upregulate CD8 and CD4 Expression Upon Antigenic Stimulation

**Figure 7:**
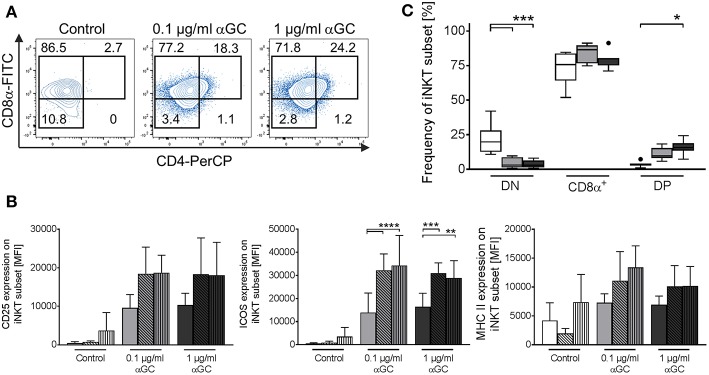
Changes in iNKT cell subset frequency upon antigenic activation. Porcine PBMC were incubated in the presence of of 0.1 μg/ml or 1 μg/ml αGC or DMSO as vehicle control for 4 days. **(A)** Expression of CD8α and CD4 on iNKT cells was investigated. Representative flow cytometric plots of controls (left plot), low-dose (middle plot), and high-dose αGC (right plot) stimulated iNKT cells are shown. CD8α^−^/CD4^−^ (DN), CD8α^+^/CD4^−^ (CD8α^+^), and CD8α^+^/CD4^+^ (DP) subsets in iNKT cells were identified as previously shown. Numbers in the plot show the frequencies of the respective gate. **(B)** Frequency of iNKT cell subsets in controls (white) and after stimulation with 0.1 μg/ml (gray) or 1 μg/ml αGC (black) shown as Tukey box plots (*n* = 7). **(C)** Expression level of CD25 (left graph), ICOS (middle graph), and MHC II (right graph) on DN (empty bars), CD8α^+^ (diagonal black hatching), and DP (vertical black stripes) iNKT cells was investigated in control cells (white) and cells stimulated with 0.1 μg/ml (gray) or 1 μg/ml (black) αGC (mean (SD), *n* = 4). ^*^p < 0.05, ^**^p < 0.01, ^***^p < 0.001, ^****^p < 0.0001, repeated-measures one-way ANOVA with Holm-Sidak's *post-hoc* test for multiple comparisons.

Naïve porcine iNKT cells were mostly CD8α^+^ or DN and did not display a distinct CD4^+^ population. However, after stimulation with αGC, frequencies of iNKT cell subsets changed significantly: DN iNKT decreased from 21.5 ± 10.8% in controls to 4.1 ± 3.6% in low-dose and 4.0 ± 2.8% in high-dose αGC-stimulated samples (Figures 7A,B). At the same time, the frequency of CD8α^+^ iNKT cells increased from 73.7 ± 11.9% in controls to 84.1 ± 6.5% in low-dose and 79.6 ± 6.2% in high-dose stimulated samples (Figures 7A,B). Stimulation of porcine iNKT cells with αGC resulted in a significant increase of DP iNKT cells. In controls, only 3.6 ± 1.9% of all iNKT cells were DP. Stimulated iNKT cells upregulated CD4 expression, resulting in 11.2 ± 4.4% DP iNKT cells in low-dose and 15.5 ± 5.2% DP iNKT cells in high-dose αGC-stimulated samples (Figures 7A,B). Whether these subsets have functional implications was analyzed by investigation of CD25, ICOS, and MHC II expression on DN, CD8α^+^ and DP iNKT cells. All three proteins tended to be expressed in higher levels on CD8α^+^ and DP iNKT cells, while expression levels on DN iNKT cells were always the lowest (Figure 7C). However, this was statistically significant only for ICOS expression.

### Porcine iNKT Cells Express IFNγ, Upregulate Perforin, and Display Fast Degranulation Upon Antigenic Activation

Among the effector mechanisms of iNKT cells are the secretion of effector cytokines and cytotoxicity. Porcine iNKT cells have been shown to secrete IFNγ upon unspecific stimulation with phorbol myristate acetate (PMA) and ionomycin (36, 37). Knowledge about antigen-specific induction of effector molecule production in porcine iNKT cells is missing. Therefore, we investigated expression of IFNγ and perforin and the cytolytic capacities of cTC and iNKT cells after stimulation of porcine PBMC with αGC.

**Figure 8:**
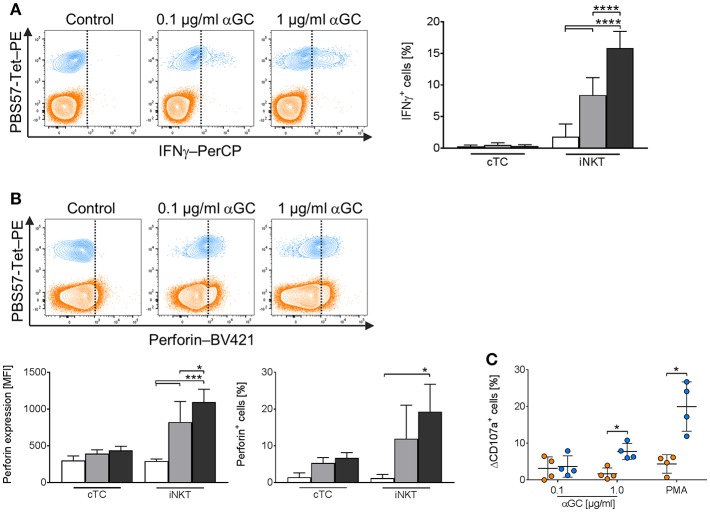
Expression of IFNγ and perforin and degranulation of activated porcine iNKT cells upon antigenic activation. Porcine PBMC were cultivated in the presence of 0.1 μg/ml (gray) or 1 μg/ml αGC (black) or DMSO (white) as a control for 4 days. At day 4, the cells were restimulated with medium containing the respective treatment. 2 h later, Brefeldin A was added and the cells were incubated for another 4 h. Intracellular expression of IFNg and perforin in iNKT cells (blue) and cTC (orange) was analyzed by flow cytometry. **(A)** Representative flow cytometric plots showing IFNγ expression in iNKT cells and cTC (vertical line indicates threshold based on expression level in controls). Frequency of IFNγ-expressing iNKT cells and cTC in controls and after stimulation with 0.1 μg/ml or 1 μg/ml αGC (*n* = 5-7). **(B)** Representative flow cytometric plots showing perforin expression (vertical line indicates threshold based on expression level in controls). Expression level of perforin in iNKT cells and cTC in controls and after antigenic stimulation. Perforin^+^ iNKT cells and cTC after antigenic stimulation (*n* = 4). **(C)** CD107a surface expression on iNKT cells and cTC after stimulation with 0.1 μg/ml or 1 μg/ml αGC and PMA/ionomycin. Specific degranulation was calculated as the difference in surface expression of stimulated and control cells and is given as ΔCD107a (*n* = 4). Data shown as mean (SD), ^*^p < 0.05, ^***^p < 0.001, ^****^p < 0.0001, repeated-measures one-way ANOVA with Holm-Sidak's *post-hoc* test for multiple comparisons.

iNKT cells showed a dose-dependent increase of IFNγ expression. Treatment of PBMC with low-dose αGC resulted in 8.4% IFNγ^+^ iNKT cells, while high-dose αGC resulted in 15.9% IFNγ^+^ iNKT cells (Figure 8A). Perforin expression (Figure 8B) increased significantly in iNKT cells upon treatment with both low- and high-dose αGC (MFI: 822.3 ± 280.3 vs. 1,095 ± 175, respectively) in contrast to naïve cells (MFI: 294 ± 33). In contrast, cTC did not express IFNγ (Figure 8A) after stimulation with low- and high-dose αGC. There was, however, an increased frequency of perforin^+^ cTC (Figure 8B) after stimulation with αGC. Porcine iNKT cells also displayed cytolytic capacities in response to αGC stimulation. While low-dose αGC did not result in significant changes in CD107a expression on iNKT cells or cTC, high-dose αGC resulted in a significantly higher frequency of CD107a^+^ iNKT cells (Figure 8C). Unspecific stimulation with PMA/ionomycin resulted in an even higher frequency of CD107a^+^ iNKT cells. However, cTC did not show significant changes of CD107a expression. Thus, we demonstrated antigen-specific induction of the effector molecules IFNγ and perforin and degranulation of porcine iNKT cells.

### iNKT Cell Frequency in Swine Decreases With Age

**Figure 9:**
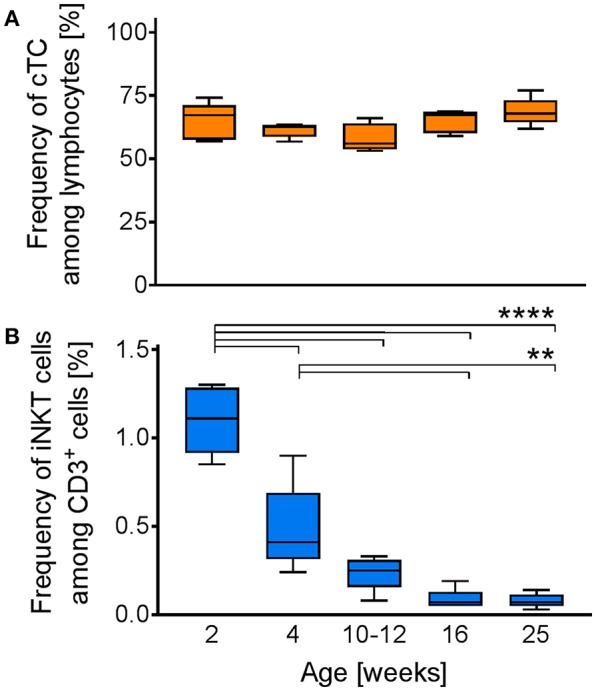
Age-dependent changes in iNKT cell frequency. Whole blood from swine of the indicated age (*n* = 5) was investigated for the frequencies of iNKT cells and cTC among CD3^+^ lymphocytes. Box plots showing the frequency of **(A)** CD3^+^ cells among lymphocytes and **(B)** iNKT cells among CD3^+^ cells. ^**^p < 0.01, ^****^p < 0.0001, ordinary one-way ANOVA with Holm-Sidak's *post-hoc* test for multiple comparisons.

Previous studies in humans indicated an important role for iNKT cells in early stages of life, evidenced by a higher percentage of iNKT cells in young individuals. To investigate whether the iNKT cell frequency in swine is also age-dependent, we analyzed the percentage of CD3^+^ cells among lymphocytes (Figure 9A) and iNKT cells among CD3^+^ lymphocytes (Figure 9B) in blood of healthy pigs two-weeks, four-weeks, 10-12-weeks, 16-weeks, and 25-weeks of age. There was no difference in the frequency of CD3^+^ cells among the different groups. In contrast, we found the frequency of iNKT cells in 2-week-old piglets (1.1% ± 0.19) to be significantly higher than in all other groups. iNKT cell frequency declined rapidly with increasing age. Four-week-old piglets still displayed significantly higher proportions of iNKT cells (0.48% ± 0.25) than older animals. The frequency of iNKT cells was still elevated, but not statistically significant, in 10–12 week-old pigs (0.24% ± 0.1) compared to older animals. In comparison to 2-week-old piglets, iNKT cell frequency was markedly reduced. In 16-week-old and 25-week-old pigs, iNKT cell frequency remained on a comparable low level (0.09% ± 0.06, 0.08% ± 0.04, respectively).

### Virus-Infected Swine Display Increasing Frequencies of iNKT Cells in Disease-Related Tissues

**Figure 10:**
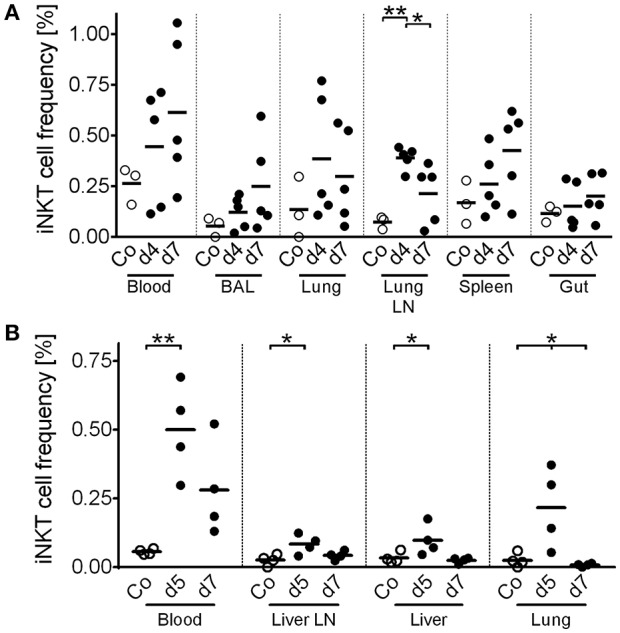
*In vivo* dynamics of porcine iNKT cells during viral infections. Pigs were experimentally infected with **(A)** IAV (*n* = 3-5) or **(B)** ASFV (*n* = 4). At the indicated time after infection, animals were euthanized and lymphocytes of the indicated tissues were isolated. Frequency of iNKT among CD3^+^ lymphocytes was then investigated using flow cytometry. Open circles show uninfected control animals (Co). Closed circles show infected animals at the indicated time post infection. Each symbol represents an individual animal with a line indicating mean. BAL, Broncho alveolar lavage. Lung LN, lung lymph node (*Nodi lymphatici tracheobronchales inferiores*). Liver LN, liver lymph node (*Nodi lymphatici hepatici*). ^*^p < 0.05, ^**^p < 0.01, ordinary one-way ANOVA with Holm-Sidak's *post-hoc* test for multiple comparisons.

iNKT cells are among the first responders after microbial infection. In pigs, iNKT cell dynamics upon infection have not been investigated so far. Hence, we measured iNKT cell frequency in viral infections of either high zoonotic potential, i.e., IAV (H1N1; Figure 10A), or of high veterinary and economic importance, i.e., ASFV strain Armenia08 (Figure 10B). Over the course of the study, IAV-infected animals showed no clinical signs of disease. However, during subclinical IAV infection (Figure 10A), we found a significant increase in iNKT cell frequency in lung lymph nodes (*Nodus lymphaticus tracheobronchales inferiores*) at 4 days post infection (dpi), which decreased until 7 dpi to levels still higher than in control animals. In line with this finding, iNKT cell frequencies tended to increase non-significantly in peripheral blood, broncho-alveolar lavage (BAL), and lung at 4 dpi, which returned to control levels at 7 dpi in all tissues. In spleen, iNKT cell frequency peaked at 7 dpi. There were no changes in iNKT cell frequency in the gut. In contrast to IAV, ASFV-infected swine showed typical clinical signs of severe disease. ASFV infection was fatal in all animals in this study. During ASFV infection, we detected a significant increase in iNKT cell frequency in blood and lung at 5 dpi. In the lung, frequency dropped to control levels at 7 dpi, while they remained elevated, although not significantly, in blood. iNKT cell frequency was also increased in liver and one of the liver lymph nodes (*Nodi lymphatici hepatici*) 5 dpi but was on control levels again at 7 dpi. We therefore described the first iNKT cell dynamics in virus-infected swine.

**Figure 11:**
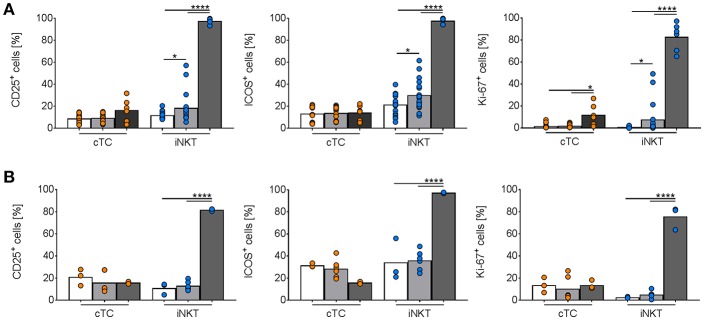
*In vitro* activation of porcine iNKT cells during viral infections. Freshly isolated porcine PBMC were incubated without stimulation (white bars) or in the presence of supernatant of purified CD172a^+^ cells infected with **(A)** IAV (MOI 1; gray) or **(B)** ASFV (MOI 0.1; gray), or 1 μg/ml αGC (black) for 4 days. Frequencies of iNKT cells (blue circles) and cTC (orange circles) positive for CD25, ICOS, and Ki-67 are shown. Pooled data of two experiments with six individual pigs for IAV and three individual pigs for ASFV shown as mean (SD), ^*^p < 0.05, ^****^p < 0.0001, ordinary one-way ANOVA with Holm-Sidak's *post-hoc* test for multiple comparisons.

To further evaluate the role of porcine iNKT cells in the aforementioned viral infections and to test our findings with the synthetic CD1d ligand αGC, we stimulated porcine PBMC with supernatant of CD172a^+^ cells infected with IAV or ASFV. We found a small but significant increase of CD25^+^, ICOS^+^, and Ki-67^+^ iNKT cells after stimulation with IAV-conditioned supernatant (Figure 11A). For ASFV, there was no detectable activation of iNKT cells (Figure 11B).

## Discussion

iNKT cells are a subset of innate lymphocytes located at potential pathogen entry sites at mucosal surfaces and lymphoid tissues. Even though they are a rare population in the vast pool of lymphocytes, they are pivotal orchestrators of innate and adaptive responses (58–60). Because of this central role in immunity, iNKT cells and their cellular responses have been investigated extensively in mice and humans. However, a large animal model for immunological research in general and iNKT cell research in specific is still needed. Because of their physiological and immunological similarities with humans, pigs exhibit high potential as a biomedical model for infectious diseases (3–10). Moreover, because pigs are of high veterinary and economic importance, understanding of their immune system has an invaluable relevance exceeding mere scientific modeling.

We comprehensively characterized peripheral porcine iNKT cells and provided evidence for similarities with their human and murine counterparts. Naïve peripheral iNKT cells in swine were predominantly CD8α^+^ and did not display a distinct CD4^+^ population, which confirms earlier data (36, 37). These characteristics are comparable to human peripheral iNKT cells, which are predominantly CD8α^+^ or DN, and in contrast to murine iNKT cells, which are mostly CD4^+^ and lack CD8 surface expression (61). CD8α^+^ iNKT cells in swine and humans are principally CD8αα^+^ but do not express the CD8 β-chain (61). Differences in the study design in various species may hamper accurate comparative analysis of iNKT. Human and murine iNKT are often expanded *in vitro* before experimental investigations, resulting in significant changes of the subset distribution (61, 62). Therefore, some authors question the reliability of functional classifications of CD4/CD8 subsets in iNKT. We investigated freshly isolated iNKT. Hence, the described characteristics of iNKT subsets in this study are largely unaffected by *ex vivo* sample processing.

Porcine iNKT cells displayed an antigen-experienced phenotype, indicated by lack of CD45RA expression on the cell surface. Applying the characterization of human memory T cells to swine, porcine CCR7^+^/CD27^+^ iNKT cells represent a subset functionally similar to central memory cells, while CCR^−^/CD27^+^ and CCR^−^/CD27^−^ iNKT cells resemble transitional memory cells and effector memory cells, respectively (56). Upon activation, central memory cells produce IL-2 and rapidly proliferate. After differentiation into effector memory cells, they express cytokines, including IFNγ (55). An elevated fraction of CD27^−^/CCR7^+^ iNKT cells, probably representing activated effector cells with *de novo* expression of CCR7 (63), further strengthens our hypothesis that iNKT cells exhibit a preactivated phenotype in naïve animals. A comparable phenotype has been shown in human and murine iNKT cells as well (13, 64–66). This preactivation is discussed as the result of a lower activation threshold of iNKT cells in comparison to cTC (67) or exposition to endogenous ligands (68). Either way, preactivation of iNKT cells *in vivo*, in line with preformed cytokine mRNA (13), additionally emphasizes the ability of iNKT cells to respond immediately to stimuli (66). About half of all porcine iNKT cells expressed CCR7, which is critical for T cell extravasation and migration into T cell areas of secondary lymphoid tissues (69). CCR7 expression on porcine peripheral iNKT cells could therefore also explain the significant *in vivo* increases of iNKT cell frequencies in regional lymph nodes during infection with IAV (H1N1) as well as with ASFV strain Armenia08. Functional CCR7 expression, i.e., chemotactic migration to CCR7 ligands, has been shown for peripheral murine and human iNKT cells (70, 71). Moreover, CCR7 seems to be pivotal for the differentiation into effector subsets in the periphery (71). This indicates that porcine CCR7^+^ iNKT cells are licensed to early migration from peripheral blood to secondary lymphoid tissues as well. Additionally, we found high levels of CD27 on porcine CD8α^+^ iNKT cells but not on DN iNKT cells. CD27 is essential for survival of CD8^+^ effector cells, especially after multiple rounds of cell division (72, 73). Ligand-binding of CD27 on CD8^+^ cells induces proliferation even in the absence of *bona fide* stimuli such as IL-2 (74). Cumulatively, our results emphasize that porcine iNKT cells display an effector-memory phenotype and that activation of porcine CD8α^+^ iNKT cells may occur in the absence of co-stimulation by other cells.

Our phenotypic characterization further indicated the presence of two main iNKT subsets, iNKT1 and iNKT2, and a minor subset, non-iNKT1/2. According to studies in mice, these subsets differ not only phenotypically but also functionally. iNKT1 exhibit properties associated with Th1 cells, like production of IFNγ, while iNKT2 produce the Th2 cytokine IL-4. The non-iNKT1/2 fraction may contain several other iNKT cell subsets, which at present cannot be further investigated in swine due to lack of detection systems. Among the possible iNKT subsets are iNKT17, producing IL-17, and regulatory and follicular helper iNKT cells. Different regulatory iNKT cells have been identified by the expression of FoxP3 or E4BP4 in mice (75, 76), follicular helper iNKT have been described as Bcl-6^+^ (19). A recent study has provided evidence indicating that porcine iNKT cells also provide non-cognate B cell help (32), indicating that follicular helper iNKT cells exist in pigs as well. In humans, CD4^+^ iNKT cells are the most efficient B cell helpers (77). Upregulation of CD4 and MHC II in activated porcine iNKT cells suggests that CD4^+^ iNKT cells in swine have similar functions. MHC II expression by human cTC and iNKT cells is also upregulated upon activation during viral infections (78, 79). Both cell types may act as effective Antigen-presenting cells (APC) (52). Expression of MHC II on the surface of T lymphocytes has also been shown for a variety of other species, including rats, canine, bovine, and equine (52). Murine T cells, unlike cells from other mammals, do not express MHC II on their own but rather acquire it from other cells (52). We found that activation of porcine iNKT cells with αGC leads to abundant cell surface expression of MHC II. Thus, porcine iNKT cells reflect human iNKT cells better than murine iNKT cells. However, the role of MHC II^+^ iNKT cells is not entirely understood. Earlier studies indicated that interaction of MHC II and CD4 on CD4^+^ iNKT cells could boost previous TCR-dependent activation by *trans*-interaction of MHC II^+^ and CD4^+^ iNKT cells, respectively (80). Upregulation of CD4 as well as MHC II by activated porcine iNKT cells indicates that such a process may occur in swine as well.

Human as well as murine iNKT cells primarily display an effector phenotype (61). We showed that porcine iNKT cells have similar characteristics. Naïve porcine iNKT cells abundantly expressed CD5 and were positive for CD25, MHC II, and ICOS. These markers are associated with effector cells (49–52). Expression of CD25 on naïve iNKT cells has also been shown in humans and mice (81, 82), ensuring prompt iNKT cell responsiveness (83). Expression of CD5 is upregulated by human cTC upon activation (49). We observed a corresponding increase of CD5 on the surface of activated porcine iNKT cells, thereby demonstrating highly activated cells (49). However, the role of CD5 on cTC and iNKT cells is not entirely understood. Molecular studies with human CD5 indicate that homophilic interactions with CD5 on other T cells or APC in *trans* or on the same cell in *cis* are needed for the regulation of T cell immunity (84). CD5 could also be used for regulation of iNKT cell responses, because expression of CD5 has been shown to inhibit TCR-dependent cell activation (49). Therefore, the high levels of CD5 on activated iNKT cells could limit further activation and possible immunopathology. Responses of iNKT cells are further regulated by ICOS. Interaction of ICOS with ICOSL, expressed exclusively on APC (85), is essential for homeostasis, activation, and survival of iNKT cells (51, 86, 87). Expression of ICOS, along with the receptors for IL-12 and IL-18, is induced by PLFZ, thereby shaping the ability of iNKT cells to respond to stimuli (88). This underlines the importance of ICOS for iNKT cell functionality. Some studies indicate that ICOS expression is associated with a Th1-based response, because ICOS expression on iNKT cells correlates with a pro-inflammatory phenotype and expression of IFNγ (86, 87). Other studies suggest that ICOS is not linked to a certain Th-subset differentiation but rather identifies cells in an effector state (89). Thus, ICOS expression on porcine iNKT cells does not necessarily indicate differentiation of iNKT cells into iNKT1. However, in context with the expression of other markers, such as CD25 or CD5, their shift to a T-bet^+^ iNKT1 phenotype, and their expression of IFNγ and perforin, it seems accurate that αGC-stimulated porcine iNKT cells differentiate primarily into Th1/iNKT1.

In this study, we observed a higher expression of activation and effector markers, like CD25 and MHC II, on CD8α^+^ iNKT cells than on CD8α^−^ iNKT cells. Moreover, CD8α^+^ iNKT cells were the primary subset we detected during Th1-biased iNKT cell responses. Comparable results have been described in earlier studies, where CD8α^+^ iNKT cells produced higher amounts of IFNγ during unspecific activation with PMA/ionomycin (37). CD8α has also been described as an activation marker on another population of unconventional porcine lymphocytes, γδ T cells (90). This indicates that CD8α also acts as a marker of maturation and effector functions on porcine iNKT cells. Additionally, since activation resulted in a loss of DN iNKT cells and an increase of DP and CD8α^+^ iNKT cells, both with a significantly higher ICOS expression than DN iNKT cells, CD8α^+^ and DP iNKT cells might represent highly differentiated effector subsets. The high expression of effector markers even in steady state indicates that porcine iNKT cells are also licensed for rapid responses. Overall, porcine iNKT cells phenotypically mimic human iNKT cells significantly better than murine iNKT cells.

Porcine iNKT cells upregulated perforin expression and displayed degranulation as shown by increased CD107a surface expression upon antigenic stimulation. This feature is shared with iNKT cells in mice and humans (17, 91–93). Cytotoxicity, including perforin production, is induced independent of TCR stimulation by pro-inflammatory cytokines like IL-12 or IL-18 secreted by APC (91, 94). How iNKT cells precisely mediate cytotoxicity is still under discussion. Perforin-mediated lysis was described in viral infections and cancer models (15, 16, 91), while other studies suggested rather Fas/FasL-dependent lysis of target cells by iNKT cells (18, 94). Both pathways are CD1d-dependent, although CD1d-independent lysis mediated by NKG2D has also been described (95). Which of these pathways prevails in a specific context likely depends on the activation mode and availability of relevant molecules on the surface of target cells (14). Deciphering activation and mode of porcine iNKT cell cytotoxicity require in-depth analysis. Our phenotypic and functional characterization paves ways for such studies. The increase of perforin-expressing cTC we saw in our studies is likely due to direct and indirect effects on cTC during iNKT cell activation. IFNγ, highly expressed by activated iNKT cells, is known to enhance the cytotoxic activity of T cells by autocrine and paracrine stimulation (96). Moreover, IFNγ has been shown to induce the expression of the high-affinity IL-12 receptor (97). This enables cTC to react to IL-12, secreted by APC during αGC stimulation. IL-12 in turn is known to enhance perforin responses (98). This bystander activation provides further protection during ongoing immune responses and demonstrates the linkage of both early iNKT cell and subsequent cTC responses.

Interestingly, we only saw significant differences between the effects of the two concentrations of αGC in three cases. High-dose αGC resulted in heightened proliferation, increased perforin expression, and increased frequencies of IFNγ-producing iNKT cells. As iNKT cells do not require co-stimulation for cytokine-production (12, 13), dose-dependent effects are explained by interaction loops between APC and iNKT cells. Activated iNKT cells readily produce cytokines, like IFNγ, and induce maturation of dendritic cells, which in turn increase production of iNKT cell-stimulating cytokines, like IL-12 (99). Notably, αGC is a highly potent antigen with a high affinity to the iNKT cell TCR and a long half-life (100). Therefore, αGC likely induces stronger responses in iNKT cells than activation with natural ligands (27). The distinctive phenotype of *in vitro*-stimulated porcine iNKT cells requires *in vitro* verification with other CD1d ligands or bacterial/viral antigens and, most importantly, *in vivo* validation in infection studies.

We found a rapid age-dependent decrease in iNKT cell percentage during the first 12 weeks of life. In animals older than 12 weeks, iNKT cells maintained the same abundance. Since the frequency of CD3^+^ lymphocytes did not change, the drop in iNKT cell frequency cannot be explained by changes in the overall T cell frequency. The high iNKT cell frequencies in young individuals indicate that iNKT cells play a critical role for immunological responses in the first weeks of life. Similar age-dependent decreases are known in humans as well (101, 102). The frequency of iNKT cells in swine investigated for age-related changes was lower than in swine investigated in other experiments. This might be explained by different housing conditions of the pigs used for different experiments. The samples for investigation of age-dependent iNKT cell frequency were obtained from a pig farm with strict biosafety standards, limiting contact with pathogenic microorganisms. In contrast, animals for other experiments were kept at our institute under conventional conditions, enabling microbial colonization. In mice, iNKT cell frequencies decrease if animals are kept under germ-free conditions (103, 104). This indicates that iNKT cell homeostasis in swine is also at least partially dependent on the microbiome of the respective animal.

We described the first iNKT cell kinetics in pigs in two viral infections, IAV and ASFV. We observed increases in iNKT cell frequency in blood and mucosal tissues at early time points during both infections, in line with the well-documented relevance of this lymphocyte population at the onset of infection (58, 105). However, iNKT cell dynamics changed at later stages of infection and their frequencies decreased over time. Moreover, we detected small but significant activation of iNKT cells in *in vitro* assays using IAV-conditioned supernatant. A host-beneficial expansion of iNKT cells with antiviral properties during IAV infection has been described in other species as well [reviewed in Crosby and Kronenberg (27)]. Mechanistically, iNKT cells act in various ways during IAV infection. Via secreted IFNγ, iNKT cells activate multiple bystander cells and induce protective adaptive responses. Moreover, iNKT cells facilitate conventional antiviral CD8^+^ T cell responses (106), inhibit immunosuppression by myeloid-derived suppressor cells (107), and produce cytokines critical for mucosal immunity and integrity, like IL-22 (108). The iNKT cell influx during IAV infection in swine peaked significantly in the lung lymph nodes and non-significantly in lung and broncho-alveolar lavage in our study. Comparable expansions of iNKT populations in these tissues were shown during IAV infection in humans (27). This, in connection with our *in vitro* data, indicates that iNKT cells in swine may play a similar role during respiratory infections as in humans. Future studies will clarify precisely how iNKT cells are involved in antiviral immunity against IAV in swine. In response to ASFV infections, CD8^+^ T cells seem to play a major role (109). Moreover, higher IFNγ production correlated with higher protection from ASFV challenge (110). A role for iNKT cell-like cells in the immunity against ASFV infection has been discussed earlier, as cells with a NKT-like phenotype (CD3^+^/CD4^−^/CD8^+^/CD5^±^/CD6^−^/CD11b^+^/CD16^+^) expanded after co-culture of porcine PBMC with ASFV *in vitro* (110). However, as others and we showed, the phenotype of these cells resembled real iNKT cells only rudimentarily. It is therefore questionable whether the described expansion and effects are attributable to iNKT cells. Contrary to the changes *in vivo*, we did not see any ASFV-induced iNKT cell activation *in vitro*. This might be explained by immune evasion mechanisms used by pathogenic ASFV strains, that were recently been shown to block type I IFN responses in infected cells (111). Since type I IFN are potent inductors of iNKT cell activation (14), blockage of type I IFN expression would impair iNKT cell activation. However, these evasion mechanisms might at least partially be counteracted *in vivo*, which cannot be simulated *in vitro*. Our *in vivo* data still indicates that iNKT cells are involved in the immune response against ASFV because the iNKT cell frequency increased locally in affected tissues and systemically in peripheral blood. However, an immunopathological role of iNKT cells during ASFV infection cannot be excluded. Overactivation of iNKT cells may result in a cytokine storm, which further weakens the animals and contributes to morbidity (112, 113). Furthermore, excessive local responses result in immunopathology, like liver damage (114, 115), as typically seen in animals succumbed to ASFV infection. The role of porcine iNKT cells in ASFV infections has to be evaluated in future studies.

iNKT cells are increasingly coming to the fore as target cells for novel vaccine adjuvants. New and more effective vaccines against infectious diseases are needed for swine as well as for humans. This is especially true for zoonotic diseases, like IAV, for which humans and swine are susceptible. αGC or αGC-analogs have been shown to induce high IgG and IgA titers against co-administered proteins in mice (116) as well as in swine (28, 29, 31). Expression of IFNγ by iNKT cells is crucial for B cell help and induction of class switch (20). We observed IFNγ expression by porcine iNKT cells upon antigenic stimulation, which indicates that pathways similar to those in mice are used by porcine iNKT cells to help B cells. αGC-adjuvanted vaccines also prime and potentiate cytotoxic CD8 T cell responses in mice (116, 117) and non-human primates (118). Targeting iNKT cells during vaccination represents a promising new way to increase vaccine efficacy. However, cellular and molecular interactions of porcine iNKT cells with effector cells of the adaptive immune system need further evaluation.

Taken together, we established a multicolor flow cytometry platform for analysis of porcine iNKT cells. Our study pioneered detailed phenotyping and differentiation of porcine iNKT cells and their effector molecules. Moreover, we provided first insights into the relevance of iNKT cells in viral diseases in pigs. We demonstrated that porcine iNKT cells display striking phenotypic and functional similarities to human but less to murine iNKT cells. Therefore, pigs were shown to be a valuable large animal model for immunological studies, especially for, but not limited to, iNKT cell research.

## Ethics Statement

All animal experiments were approved by the ethics committee of the State Office for Agriculture, Food Safety and Fishery in Mecklenburg-Western Pomerania (LALFF M-V) with reference numbers 7221.3-1-035/17 for IAV and 7221.3-1.1-064/17 for ASFV. All applicable animal welfare regulations, including EU Directive 2010/63/EC and institutional guidelines, were taken into consideration.

## Author Contributions

AS and UB: conceived and designed *in vitro* experiments. TS, TM, SB, CS, and UB: conceived and designed animal experiments. AS, JH, TS, SB, and CS: acquired animal samples. AS: performed *in vitro* experiments. AS, AD, TM, and UB: data analysis and interpretation. AS and UB: manuscript preparation. All authors reviewed and approved the final version of the manuscript.

### Conflict of Interest Statement

The authors declare that the research was conducted in the absence of any commercial or financial relationships that could be construed as a potential conflict of interest.

## Supplementary Material

Supplementary Table 1 Antibodies used in this study.

## References

[1] Gutierrez K Dicks N Glanzner WG Agellon LB Bordignon V. Efficacy of the porcine species in biomedical research. Front Genet. (2015) 6:293. 10.3389/fgene.2015.00293 26442109 PMC4584988

[2] Seok J Warren HS Cuenca AG Mindrinos MN Baker HV Xu W. Genomic responses in mouse models poorly mimic human inflammatory diseases. Proc Natl Acad Sci USA. (2013) 110:3507–12. 10.1073/pnas.1222878110 23401516 PMC3587220

[3] Fairbairn L Kapetanovic R Sester DP Hume DA. The mononuclear phagocyte system of the pig as a model for understanding human innate immunity and disease. J Leukoc Biol. (2011) 89:855–71. 10.1189/jlb.1110607 21233410

[4] Giraud S Favreau F Chatauret N Thuillier R Maiga S Hauet T. Contribution of large pig for renal ischemia-reperfusion and transplantation studies: the preclinical model. J Biomed Biotechnol. (2011) 2011:532127. 10.1155/2011/532127 21403881 PMC3051176

[5] Mair KH Sedlak C Kaser T Pasternak A Levast B Gerner W. The porcine innate immune system: an update. Dev Comp Immunol. (2014) 45:321–43. 10.1016/j.dci.2014.03.022 24709051 PMC7103209

[6] Swindle MM Makin A Herron AJ Clubb FJ Jr Frazier KS. Swine as models in biomedical research and toxicology testing. Vet Pathol. (2012) 49:344–56. 10.1177/0300985811402846 21441112

[7] Renukaradhya GJ Manickam C Khatri M Rauf A Li X Tsuji M. Functional invariant NKT cells in pig lungs regulate the airway hyperreactivity: a potential animal model. J Clin Immunol. (2011) 31:228–39. 10.1007/s10875-010-9476-4 21042929 PMC4450678

[8] Ugolini M Gerhard J Burkert S Jensen KJ Georg P Ebner F. Recognition of microbial viability via TLR8 drives TFH cell differentiation and vaccine responses. Nat Immunol. (2018) 19:386–96. 10.1038/s41590-018-0068-4 29556002

[9] Maisonnasse P Bouguyon E Piton G Ezquerra A Urien C Deloizy C. The respiratory DC/macrophage network at steady-state and upon influenza infection in the swine biomedical model. Mucosal Immunol. (2016) 9:835–49. 10.1038/mi.2015.105 26530136

[10] Meurens F Summerfield A Nauwynck H Saif L Gerdts V. The pig: a model for human infectious diseases. Trends Microbiol. (2012) 20:50–7. 10.1016/j.tim.2011.11.002 22153753 PMC7173122

[11] Iwasaki A Foxman EF Molony RD. Early local immune defences in the respiratory tract. Nat Rev Immunol. (2017) 17:7–20. 10.1038/nri.2016.117 27890913 PMC5480291

[12] Uldrich AP Crowe NY Kyparissoudis K Pellicci DG Zhan Y Lew AM. NKT cell stimulation with glycolipid antigen *in vivo*: costimulation-dependent expansion, Bim-dependent contraction, and hyporesponsiveness to further antigenic challenge. J Immunol. (2005) 175:3092–101. 10.4049/jimmunol.175.5.3092 16116198 PMC1360163

[13] Stetson DB Mohrs M Reinhardt RL Baron JL Wang ZE Gapin L. Constitutive cytokine mRNAs mark natural killer (NK) and NK T cells poised for rapid effector function. J Exp Med. (2003) 198:1069–76. 10.1084/jem.20030630 14530376 PMC2194220

[14] Kohlgruber AC Donado CA LaMarche NM Brenner MB Brennan PJ. Activation strategies for invariant natural killer T cells. Immunogenetics. (2016) 68:649–63. 10.1007/s00251-016-0944-8 27457886 PMC5745583

[15] Bassiri H Das R Guan P Barrett DM Brennan PJ Banerjee PP. iNKT cell cytotoxic responses control T-lymphoma growth *in vitro* and *in vivo*. Cancer Immunol Res. (2014) 2:59–69. 10.1158/2326-6066.CIR-13-0104 24563871 PMC3927984

[16] Bassiri H Das R Nichols KE. Invariant NKT cells: killers and conspirators against cancer. Oncoimmunology. (2013) 2:e27440. 10.4161/onci.27440 24575380 PMC3926875

[17] Gumperz JE Miyake S Yamamura T Brenner MB. Functionally distinct subsets of CD1d-restricted natural killer T cells revealed by CD1d tetramer staining. J Exp Med. (2002) 195:625–36. 10.1084/jem.20011786 11877485 PMC2193772

[18] Wingender G Krebs P Beutler B Kronenberg M. Antigen-specific cytotoxicity by invariant NKT cells *in vivo* is CD95/CD178-dependent and is correlated with antigenic potency. J Immunol. (2010) 185:2721–9. 10.4049/jimmunol.1001018 20660713 PMC2989418

[19] Chang PP Barral P Fitch J Pratama A Ma CS Kallies A. Identification of Bcl-6-dependent follicular helper NKT cells that provide cognate help for B cell responses. Nat Immunol. (2011) 13:35–43. 10.1038/ni.2166 22120117

[20] Doherty DG Melo AM Moreno-Olivera A Solomos AC. Activation and regulation of B cell responses by invariant natural killer T cells. Front Immunol. (2018) 9:1360. 10.3389/fimmu.2018.01360 29967611 PMC6015876

[21] Benlagha K Kyin T Beavis A Teyton L Bendelac A. A thymic precursor to the NK T cell lineage. Science. (2002) 296:553–5. 10.1126/science.1069017 11968185

[22] Constantinides MG Bendelac A. Transcriptional regulation of the NKT cell lineage. Curr Opin Immunol. (2013) 25:161–7. 10.1016/j.coi.2013.01.003 23402834 PMC3647452

[23] Lee YJ Holzapfel KL Zhu J Jameson SC Hogquist KA. Steady-state production of IL-4 modulates immunity in mouse strains and is determined by lineage diversity of iNKT cells. Nat Immunol. (2013) 14:1146–54. 10.1038/ni.2731 24097110 PMC3824254

[24] McNab FW Berzins SP Pellicci DG Kyparissoudis K Field K Smyth MJ. The influence of CD1d in postselection NKT cell maturation and homeostasis. J Immunol. (2005) 175:3762–8. 10.4049/jimmunol.175.6.3762 16148122

[25] Strong BS Newkold TJ Lee AE Turner LE Alhajjat AM Heusel JW. Extrinsic allospecific signals of hematopoietic origin dictate iNKT cell lineage-fate decisions during development. Sci Rep. (2016) 6:28837. 10.1038/srep28837 27354027 PMC4926280

[26] Watarai H Sekine-Kondo E Shigeura T Motomura Y Yasuda T Satoh R. Development and function of invariant natural killer T cells producing T(h)2- and T(h)17-cytokines. PLoS Biol. (2012) 10:e1001255. 10.1371/journal.pbio.1001255 22346732 PMC3274505

[27] Crosby CM Kronenberg M. Tissue-specific functions of invariant natural killer T cells. Nat Rev Immunol. (2018) 18:559–74. 10.1038/s41577-018-0034-2 29967365 PMC6343475

[28] Artiaga BL Whitener RL Staples CR Driver JP. Adjuvant effects of therapeutic glycolipids administered to a cohort of NKT cell-diverse pigs. Vet Immunol Immunopathol. (2014) 162:1–13. 10.1016/j.vetimm.2014.09.006 25441499

[29] Artiaga BL Yang G Hackmann TJ Liu Q Richt JA Salek-Ardakani S. α-Galactosylceramide protects swine against influenza infection when administered as a vaccine adjuvant. Sci Rep. (2016) 6:23593. 10.1038/srep23593 27004737 PMC4804283

[30] Artiaga BL Yang G Hutchinson TE Loeb JC Richt JA Lednicky JA. Rapid control of pandemic H1N1 influenza by targeting NKT-cells. Sci Rep. (2016) 6:37999. 10.1038/srep37999 27897246 PMC5126553

[31] Dwivedi V Manickam C Dhakal S Binjawadagi B Ouyang K Hiremath J. Adjuvant effects of invariant NKT cell ligand potentiates the innate and adaptive immunity to an inactivated H1N1 swine influenza virus vaccine in pigs. Vet Microbiol. (2016) 186:157–63. 10.1016/j.vetmic.2016.02.028 27016770

[32] Renu S Dhakal S Kim E Goodman J Lakshmanappa YS Wannemuehler MJ Intranasal delivery of influenza antigen by nanoparticles, but not NKT-cell adjuvant differentially induces the expression of B-cell activation factors in mice and swine. Cell Immunol. (2018) 329:27–30. 10.1016/j.cellimm.2018.04.005 29665972

[33] Eguchi-Ogawa T Morozumi T Tanaka M Shinkai H Okumura N Suzuki K. Analysis of the genomic structure of the porcine CD1 gene cluster. Genomics. (2007) 89:248–61. 10.1016/j.ygeno.2006.10.003 17112699

[34] Bendelac A. Positive selection of mouse NK1+ T cells by CD1-expressing cortical thymocytes. J Exp Med. (1995) 182:2091–6. 10.1084/jem.182.6.2091 7500054 PMC2192225

[35] Yang G Artiaga BL Hackmann TJ Samuel MS Walters EM Salek-Ardakani S. Targeted disruption of CD1d prevents NKT cell development in pigs. Mamm Genome. (2015) 26:264–70. 10.1007/s00335-015-9564-0 25930071 PMC4830386

[36] Thierry A Robin A Giraud S Minouflet S Barra A Bridoux F. Identification of invariant natural killer T cells in porcine peripheral blood. Vet Immunol Immunopathol. (2012) 149:272–9. 10.1016/j.vetimm.2012.06.023 22939274

[37] Yang G Artiaga BL Lewis ST Driver JP. Characterizing porcine invariant natural killer T cells: a comparative study with NK cells and T cells. Dev Comp Immunol. (2017) 76:343–51. 10.1016/j.dci.2017.07.006 28694168

[38] Yang G Richt JA Driver JP. Harnessing invariant NKT cells to improve influenza vaccines: a pig perspective. Int J Mol Sci. (2017) 19:68. 10.3390/ijms19010068 29280974 PMC5796018

[39] Yang H Binns RM. Expression and regulation of the porcine CD44 molecule. Cell Immunol. (1993) 149:117–29. 10.1006/cimm.1993.1141 8513507

[40] Yang G Artiaga BL Lomelino CL Jayaprakash AD Sachidanandam R McKenna R. Next generation sequencing of the pig alphabeta TCR repertoire identifies the porcine invariant NKT cell receptor. J Immunol. (2019) 202:1981–91. 10.4049/jimmunol.1801171 30777925 PMC6606045

[41] Spearman C The method of “right and wrong cases” (constant stimuli) without Gauss's formula. Br J Psychol. (1908) 2:227–42. 10.1111/j.2044-8295.1908.tb00176.x

[42] Kärber G Beitrag zur kollektiven Behandlung pharmakologischer Reihenversuche. Naunyn Schmiedebergs Arch Exp Pathol Pharmakol. (1931) 162:480–3. 10.1007/BF01863914

[43] Spackman E Senne DA Myers TJ Bulaga LL Garber LP Perdue ML. Development of a real-time reverse transcriptase PCR assay for type A influenza virus and the avian H5 and H7 hemagglutinin subtypes. J Clin Microbiol. (2002) 40:3256–60. 10.1128/JCM.40.9.3256-3260.2002 12202562 PMC130722

[44] Pietschmann J Guinat C Beer M Pronin V Tauscher K Petrov A. Course and transmission characteristics of oral low-dose infection of domestic pigs and European wild boar with a Caucasian African swine fever virus isolate. Arch Virol. (2015) 160:1657–67. 10.1007/s00705-015-2430-2 25916610

[45] Kitani H Yoshioka M Takenouchi T Sato M Yamanaka N. Characterization of the liver-macrophages isolated from a mixed primary culture of neonatal swine hepatocytes. Results Immunol. (2014) 4:1–7. 10.1016/j.rinim.2014.01.001 24707456 PMC3973824

[46] Fernandez CS Cameron G Godfrey DI Kent SJ. *Ex-vivo* α-galactosylceramide activation of NKT cells in humans and macaques. J Immunol Methods. (2012) 382:150–9. 10.1016/j.jim.2012.05.019 22683545

[47] Singh D Ghate M Godbole S Kulkarni S Thakar M. Functional invariant natural killer T cells secreting cytokines are associated with non-progressive human immunodeficiency virus-1 infection but not with suppressive anti-retroviral treatment. Front Immunol. (2018) 9:1152. 10.3389/fimmu.2018.01152 29881390 PMC5976739

[48] Zekavat G Mozaffari R Arias VJ Rostami SY Badkerhanian A Tenner AJ. A novel CD93 polymorphism in non-obese diabetic. (NOD) and NZB/W F1 mice is linked to a CD4+ iNKT cell deficient state. Immunogenetics. (2010) 62:397–407. 10.1007/s00251-010-0442-3 20387063 PMC2875467

[49] Domingues RG Lago-Baldaia I Pereira-Castro I Fachini JM Oliveira L Drpic D. CD5 expression is regulated during human T-cell activation by alternative polyadenylation, PTBP1, and miR-204. Eur J Immunol. (2016) 46:1490–503. 10.1002/eji.201545663 27005442 PMC5555168

[50] Waldmann TA. The multi-subunit interleukin-2 receptor. Annu Rev Biochem. (1989) 58:875–911. 10.1146/annurev.biochem.58.1.875 2673025

[51] Dong C Juedes AE Temann UA Shresta S Allison JP Ruddle NH. ICOS co-stimulatory receptor is essential for T-cell activation and function. Nature. (2001) 409:97–101. 10.1038/35051100 11343121

[52] Holling TM Schooten E van Den Elsen PJ. Function and regulation of MHC class II molecules in T-lymphocytes: of mice and men. Hum Immunol. (2004) 65:282–90. 10.1016/j.humimm.2004.01.005 15120183

[53] Larbi A Fulop T From “truly naive” to “exhausted senescent” T cells: when markers predict functionality. Cytometry A. (2014) 85:25–35. 10.1002/cyto.a.22351 24124072

[54] Okada R Kondo T Matsuki F Takata H Takiguchi M. Phenotypic classification of human CD4+ T cell subsets and their differentiation. Int Immunol. (2008) 20:1189–99. 10.1093/intimm/dxn075 18635582

[55] Sallusto F Geginat J Lanzavecchia A. Central memory and effector memory T cell subsets: function, generation, and maintenance. Annu Rev Immunol. (2004) 22:745–63. 10.1146/annurev.immunol.22.012703.104702 15032595

[56] Mahnke YD Brodie TM Sallusto F Roederer M Lugli E. The who's who of T-cell differentiation: human memory T-cell subsets. Eur J Immunol. (2013) 43:2797–809. 10.1002/eji.201343751 24258910

[57] Soares A Govender L Hughes J Mavakla W de Kock M Barnard C. Novel application of Ki67 to quantify antigen-specific *in vitro* lymphoproliferation. J Immunol Methods. (2010) 362:43–50. 10.1016/j.jim.2010.08.007 20800066 PMC2989440

[58] Brigl M Bry L Kent SC Gumperz JE Brenner MB. Mechanism of CD1d-restricted natural killer T cell activation during microbial infection. Nat Immunol. (2003) 4:1230–7. 10.1038/ni1002 14578883

[59] Liew PX Kubes P. Intravital imaging - dynamic insights into natural killer T cell biology. Front Immunol. (2015) 6:240. 10.3389/fimmu.2015.00240 26042123 PMC4438604

[60] Metelitsa LS. Anti-tumor potential of type-I NKT cells against CD1d-positive and CD1d-negative tumors in humans. Clin Immunol. (2011) 140:119–29. 10.1016/j.clim.2010.10.005 21095162 PMC3444285

[61] Garner LC Klenerman P Provine NM. Insights into mucosal-associated invariant T cell biology from studies of invariant natural killer T cells. Front Immunol. (2018) 9:1478. 10.3389/fimmu.2018.01478 30013556 PMC6036249

[62] Chan AC Leeansyah E Cochrane A d'Udekem d'Acoz Y Mittag D Harrison LC. *Ex-vivo* analysis of human natural killer T cells demonstrates heterogeneity between tissues and within established CD4(+) and CD4(-) subsets. Clin Exp Immunol. (2013) 172:129–37. 10.1111/cei.12045 23480193 PMC3719939

[63] Sallusto F Kremmer E Palermo B Hoy A Ponath P Qin S. Switch in chemokine receptor expression upon TCR stimulation reveals novel homing potential for recently activated T cells. Eur J Immunol. (1999) 29:2037–45. 10.1002/(SICI)1521-4141(199906)29:06<2037::AID-IMMU2037>3.0.CO;2-V 10382767

[64] D'Andrea A Goux D De Lalla C Koezuka Y Montagna D Moretta A. Neonatal invariant Vα24+ NKT lymphocytes are activated memory cells. Eur J Immunol. (2000) 30:1544–50. 10.1002/1521-4141(200006)30:6<1544::AID-IMMU1544>3.0.CO;2-I 10898489

[65] Park SH Benlagha K Lee D Balish E Bendelac A. Unaltered phenotype, tissue distribution and function of Vα14(+) NKT cells in germ-free mice. Eur J Immunol. (2000) 30:620–5. 10.1002/1521-4141(200002)30:2<620::AID-IMMU620>3.0.CO;2-4 10671219

[66] Cole SL Benam KH McMichael AJ Ho LP. Involvement of the 4–1BB/4–1BBL pathway in control of monocyte numbers by invariant NKT cells. J Immunol. (2014) 192:3898–907. 10.4049/jimmunol.1302385 24639347

[67] van den Heuvel MJ Garg N Van Kaer L Haeryfar SM. NKT cell costimulation: experimental progress and therapeutic promise. Trends Mol Med. (2011) 17:65–77. 10.1016/j.molmed.2010.10.007 21087900 PMC3616392

[68] Van Rhijn I Kasmar A de Jong A Gras S Bhati M Doorenspleet ME. A conserved human T cell population targets mycobacterial antigens presented by CD1b. Nat Immunol. (2013) 14:706–13. 10.1038/ni.2630 23727893 PMC3723453

[69] Kim CH Johnston B Butcher EC Trafficking machinery of NKT cells: shared and differential chemokine receptor expression among Vα24+Vβ11+ NKT cell subsets with distinct cytokine-producing capacity. Blood. (2002) 100:11–6. 10.1182/blood-2001-12-0196 12070001

[70] Johnston B Kim CH Soler D Emoto M Butcher EC Differential chemokine responses and homing patterns of murine TCRα*β* NKT cell subsets. J Immunol. (2003) 171:2960–9. 10.4049/jimmunol.171.6.2960 12960320

[71] Wang H Hogquist KA. CCR7 defines a precursor for murine iNKT cells in thymus and periphery. Elife. (2018) 7:e34793. 10.7554/eLife.34793 30102153 PMC6115192

[72] Hendriks J Gravestein LA Tesselaar K van Lier RA Schumacher TN Borst J. CD27 is required for generation and long-term maintenance of T cell immunity. Nat Immunol. (2000) 1:433–40. 10.1038/80877 11062504

[73] Hendriks J Xiao Y Borst J. CD27 promotes survival of activated T cells and complements CD28 in generation and establishment of the effector T cell pool. J Exp Med. (2003) 198:1369–80. 10.1084/jem.20030916 14581610 PMC2194245

[74] Carr JM Carrasco MJ Thaventhiran JE Bambrough PJ Kraman M Edwards AD. CD27 mediates interleukin-2-independent clonal expansion of the CD8+ T cell without effector differentiation. Proc Natl Acad Sci USA. (2006) 103:19454–9. 10.1073/pnas.0609706104 17159138 PMC1697827

[75] Lynch L Michelet X Zhang S Brennan PJ Moseman A Lester C. Regulatory iNKT cells lack expression of the transcription factor PLZF and control the homeostasis of T(reg) cells and macrophages in adipose tissue. Nat Immunol. (2015) 16:85–95. 10.1038/ni.3047 25436972 PMC4343194

[76] Monteiro M Almeida CF Caridade M Ribot JC Duarte J Agua-Doce A. Identification of regulatory Foxp3+ invariant NKT cells induced by TGF-β. J Immunol. (2010) 185:2157–63. 10.4049/jimmunol.1000359 20639482

[77] Zeng SG Ghnewa YG O'Reilly VP Lyons VG Atzberger A Hogan AE. Human invariant NKT cell subsets differentially promote differentiation, antibody production, and T cell stimulation by B cells *in vitro*. J Immunol. (2013) 191:1666–76. 10.4049/jimmunol.1202223 23851681 PMC4201948

[78] Ibarrondo FJ Wilson SB Hultin LE Shih R Hausner MA Hultin PM. Preferential depletion of gut CD4-expressing iNKT cells contributes to systemic immune activation in HIV-1 infection. Mucosal Immunol. (2013) 6:591–600. 10.1038/mi.2012.101 23149661 PMC3865278

[79] Montoya CJ Catano JC Ramirez Z Rugeles MT Wilson SB Landay AL. Invariant NKT cells from HIV-1 or Mycobacterium tuberculosis-infected patients express an activated phenotype. Clin Immunol. (2008) 127:1–6. 10.1016/j.clim.2007.12.006 18304877

[80] Thedrez A de Lalla C Allain S Zaccagnino L Sidobre S Garavaglia C. CD4 engagement by CD1d potentiates activation of CD4+ invariant NKT cells. Blood. (2007) 110:251–8. 10.1182/blood-2007-01-066217 17363727

[81] Jukes JP Wood KJ Jones ND. Bystander activation of iNKT cells occurs during conventional T-cell alloresponses. Am J Transplant. (2012) 12:590–9. 10.1111/j.1600-6143.2011.03847.x 22070799 PMC3326729

[82] Schneiders FL Prodohl J Ruben JM O'Toole T Scheper RJ Bonneville M. CD1d-restricted antigen presentation by Vgamma9Vdelta2-T cells requires trogocytosis. Cancer Immunol Res. (2014) 2:732–40. 10.1158/2326-6066.CIR-13-0167 24934445

[83] Ladd M Sharma A Huang Q Wang AY Xu L Genowati I. Natural killer T cells constitutively expressing the interleukin-2 receptor alpha chain early in life are primed to respond to lower antigenic stimulation. Immunology. (2010) 131:289–99. 10.1111/j.1365-2567.2010.03304.x 20545784 PMC2967274

[84] Brown MH Lacey E. A ligand for CD5 is CD5. J Immunol. (2010) 185:6068–74. 10.4049/jimmunol.0903823 20952682 PMC2996635

[85] Wikenheiser DJ Stumhofer JS ICOS co-stimulation: friend or foe? Front Immunol. (2016) 7:304 10.3389/fimmu.2016.00304 27559335 PMC4979228

[86] Akbari O Stock P Meyer EH Freeman GJ Sharpe AH Umetsu DT. ICOS/ICOSL interaction is required for CD4+ invariant NKT cell function and homeostatic survival. J Immunol. (2008) 180:5448–56. 10.4049/jimmunol.180.8.5448 18390727 PMC2835525

[87] Kaneda H Takeda K Ota T Kaduka Y Akiba H Ikarashi Y. ICOS costimulates invariant NKT cell activation. Biochem Biophys Res Commun. (2005) 327:201–7. 10.1016/j.bbrc.2004.12.004 15629449

[88] Gleimer M von Boehmer H Kreslavsky T. PLZF controls the expression of a limited number of genes essential for NKT cell function. Front Immunol. (2012) 3:374. 10.3389/fimmu.2012.00374 23267359 PMC3528072

[89] Burmeister Y Lischke T Dahler AC Mages HW Lam KP Coyle AJ. ICOS controls the pool size of effector-memory and regulatory T cells. J Immunol. (2008) 180:774–82. 10.4049/jimmunol.180.2.774 18178815

[90] Stepanova K Sinkora M. The expression of CD25, CD11b, SWC1, SWC7, MHC-II, and family of CD45 molecules can be used to characterize different stages of gammadelta T lymphocytes in pigs. Dev Comp Immunol. (2012) 36:728–40. 10.1016/j.dci.2011.11.003 22100879

[91] Dao T Mehal WZ Crispe IN. IL-18 augments perforin-dependent cytotoxicity of liver NK-T cells. J Immunol. (1998) 161:2217–22. 9725214

[92] Ichikawa T Negishi Y Shimizu M Takeshita T Takahashi H. α-Galactosylceramide-activated murine NK1.1(+) invariant-NKT cells in the myometrium induce miscarriages in mice. Eur J Immunol. (2016) 46:1867–77. 10.1002/eji.201545923 27198610 PMC5089647

[93] Van Der Vliet HJ Nishi N Koezuka Y Peyrat MA Von Blomberg BM Van Den Eertwegh AJ. Effects of alpha-galactosylceramide. (KRN7000), interleukin-12 and interleukin-7 on phenotype and cytokine profile of human Vα24+ Vβ11+ T cells. Immunology. (1999) 98:557–63. 10.1046/j.1365-2567.1999.00920.x 10594688 PMC2326955

[94] Leite-De-Moraes MC Hameg A Arnould A Machavoine F Koezuka Y Schneider E. A distinct IL-18-induced pathway to fully activate NK T lymphocytes independently from TCR engagement. J Immunol. (1999) 163:5871–6. 10570271

[95] Kuylenstierna C Bjorkstrom NK Andersson SK Sahlstrom P Bosnjak L Paquin-Proulx D. NKG2D performs two functions in invariant NKT cells: direct TCR-independent activation of NK-like cytolysis and co-stimulation of activation by CD1d. Eur J Immunol. (2011) 41:1913–23. 10.1002/eji.200940278 21590763 PMC3523190

[96] Bhat P Leggatt G Waterhouse N Frazer IH. Interferon-gamma derived from cytotoxic lymphocytes directly enhances their motility and cytotoxicity. Cell Death Dis. (2017) 8:e2836. 10.1038/cddis.2017.67 28569770 PMC5520949

[97] Gollob JA Kawasaki H Ritz J. Interferon-gamma and interleukin-4 regulate T cell interleukin-12 responsiveness through the differential modulation of high-affinity interleukin-12 receptor expression. Eur J Immunol. (1997) 27:647–52. 10.1002/eji.1830270311 9079804

[98] Ebert EC. Interleukin-12 up-regulates perforin- and Fas-mediated lymphokine-activated killer activity by intestinal intraepithelial lymphocytes. Clin Exp Immunol. (2004) 138:259–65. 10.1111/j.1365-2249.2004.02614.x 15498035 PMC1809208

[99] Gottschalk C Mettke E Kurts C. The role of invariant natural killer T cells in dendritic cell licensing, cross-priming, and memory CD8(+) T cell generation. Front Immunol. (2015) 6:379. 10.3389/fimmu.2015.00379 26284065 PMC4517377

[100] Cerundolo V Silk JD Masri SH Salio M. Harnessing invariant NKT cells in vaccination strategies. Nat Rev Immunol. (2009) 9:28. 10.1038/nri2451 19079136

[101] Jing Y Gravenstein S Chaganty NR Chen N Lyerly KH Joyce S. Aging is associated with a rapid decline in frequency, alterations in subset composition, and enhanced Th2 response in CD1d-restricted NKT cells from human peripheral blood. Exp Gerontol. (2007) 42:719–32. 10.1016/j.exger.2007.01.009 17368996

[102] Patin E Hasan M Bergstedt J Rouilly V Libri V Urrutia A Natural variation in the parameters of innate immune cells is preferentially driven by genetic factors. Nat Immunol. (2018) 19:302–14. 10.1038/s41590-018-0049-7 29476184

[103] Wei B Wingender G Fujiwara D Chen DY McPherson M Brewer S. Commensal microbiota and CD8+ T cells shape the formation of invariant NKT cells. J Immunol. (2010) 184:1218–26. 10.4049/jimmunol.0902620 20048124 PMC3458428

[104] Wingender G Stepniak D Krebs P Lin L McBride S Wei B. Intestinal microbes affect phenotypes and functions of invariant natural killer T cells in mice. Gastroenterology. (2012) 143:418–28. 10.1053/j.gastro.2012.04.017 22522092 PMC3404247

[105] Crosby CM Kronenberg M. Invariant natural killer T cells: front line fighters in the war against pathogenic microbes. Immunogenetics. (2016) 68:639–48. 10.1007/s00251-016-0933-y 27368411 PMC5065929

[106] Paget C Ivanov S Fontaine J Blanc F Pichavant M Renneson J. Potential role of invariant NKT cells in the control of pulmonary inflammation and CD8+ T cell response during acute influenza A virus H3N2 pneumonia. J Immunol. (2011) 186:5590–602. 10.4049/jimmunol.1002348 21490153

[107] De Santo C Salio M Masri SH Lee LY Dong T Speak AO. Invariant NKT cells reduce the immunosuppressive activity of influenza A virus-induced myeloid-derived suppressor cells in mice and humans. J Clin Invest. (2008) 118:4036–48. 10.1172/JCI36264 19033672 PMC2582442

[108] Paget C Ivanov S Fontaine J Renneson J Blanc F Pichavant M. Interleukin-22 is produced by invariant natural killer T lymphocytes during influenza A virus infection: potential role in protection against lung epithelial damages. J Biol Chem. (2012) 287:8816–29. 10.1074/jbc.M111.304758 22294696 PMC3308738

[109] Oura CA Denyer MS Takamatsu H Parkhouse RM. *In vivo* depletion of CD8+ T lymphocytes abrogates protective immunity to African swine fever virus. J Gen Virol. (2005) 86:2445–50. 10.1099/vir.0.81038-0 16099902

[110] Takamatsu HH Denyer MS Lacasta A Stirling CM Argilaguet JM Netherton CL. Cellular immunity in ASFV responses. Virus Res. (2013) 173:110–21. 10.1016/j.virusres.2012.11.009 23201582

[111] Garcia-Belmonte R Perez-Nunez D Pittau M Richt JA Revilla Y. African swine fever virus Armenia/07 virulent strain controls IFN-beta production through cGAS-STING pathway. J Virol. (2019). 10.1128/JVI.02298-18 30918080 PMC6613762

[112] Scheuplein F Thariath A Macdonald S Truneh A Mashal R Schaub R. A humanized monoclonal antibody specific for invariant Natural Killer T. (iNKT) cells for *in vivo* depletion. PLoS ONE. (2013) 8:e76692. 10.1371/journal.pone.0076692 24086759 PMC3785425

[113] Van Kaer L Parekh VV Wu L. The response of CD1d-restricted invariant NKT cells to microbial pathogens and their products. Front Immunol. (2015) 6:226. 10.3389/fimmu.2015.00226 26029211 PMC4429631

[114] Osman Y Kawamura T Naito T Takeda K Van Kaer L Okumura K. Activation of hepatic NKT cells and subsequent liver injury following administration of α-galactosylceramide. Eur J Immunol. (2000) 30:1919–28. 10.1002/1521-4141(200007)30:7<1919::AID-IMMU1919>3.0.CO;2-3 10940881

[115] Takeda K Hayakawa Y Van Kaer L Matsuda H Yagita H Okumura K. Critical contribution of liver natural killer T cells to a murine model of hepatitis. Proc Natl Acad Sci USA. (2000) 97:5498–503. 10.1073/pnas.040566697 10792025 PMC25857

[116] Lee YS Lee KA Lee JY Kang MH Song YC Baek DJ. An alpha-GalCer analogue with branched acyl chain enhances protective immune responses in a nasal influenza vaccine. Vaccine. (2011) 29:417–25. 10.1016/j.vaccine.2010.11.005 21087689

[117] Venkataswamy MM Baena A Goldberg MF Bricard G Im JS Chan J. Incorporation of NKT cell-activating glycolipids enhances immunogenicity and vaccine efficacy of *Mycobacterium bovis bacillus* Calmette-Guerin. J Immunol. (2009) 183:1644–56. 10.4049/jimmunol.0900858 19620317 PMC2719834

[118] Padte NN Boente-Carrera M Andrews CD McManus J Grasperge BF Gettie A. A glycolipid adjuvant, 7DW8-5, enhances CD8+ T cell responses induced by an adenovirus-vectored malaria vaccine in non-human primates. PLoS ONE. (2013) 8:e78407. 10.1371/journal.pone.0078407 24205224 PMC3808339

