# Supplementary material for: Porcine Invariant Natural Killer T Cells: Functional Profiling and Dynamics in Steady State and Viral Infections
Source: Front Immunol. 2019 Jun 18;10:1380. doi: 10.3389/fimmu.2019.01380 (PMC6611438; doi:10.3389/fimmu.2019.01380)
Supplement: Supplementary Table 1 — Antibodies used in this study. [file Table_1.DOCX]

| Marker | Clone | Conjugate | Isotype | Dilution | Source | Catalog Nr. |
| --- | --- | --- | --- | --- | --- | --- |
| Ki-67 | B56 | BV421 | Mouse IgG1 | 1:40 | BD Biosciences | 562899 |
| Perforin | dG9 | BV421 | Mouse IgG2b | 1:40 | Biolegend | 308122 |
| T-bet | 4B10 | BV421 | Mouse IgG1 | 1:200 | Biolegend | 644832 |
| CD45RA | MIL13 | FITC | Mouse IgG1 | 1:100 | Bio-Rad | MCA1751F |
| CD8α | 76-2-11 | FITC | Mouse IgG2a | 1:100 | Southern Biotech | 4520-02 |
| CD278 (ICOS) | C398.4A | PE-Cy7 | Armenian hamster IgG | 1:100 | Biolegend | 313519 |
| PLZF | 9E12 | PE-Cy7 | Armenian hamster IgG | 1:200 | Biolegend | 145805 |
| CD4 | 74-12-4 | PerCP | Mouse IgG2b | 1:100 | BD Biosciences | 561474 |
| IFNγ | P2G10 | PerCP | Mouse IgG1 | 1:100 | BD Biosciences | 561481 |
| CD197 (CCR7) | 3D12 | Alexa 647 | Rat IgG2a | 1:100 | BD Biosciences | 557734 |
| CD3ε | PPT3 | APC | Mouse IgG1 | 1:500 | Southern Biotech | 4510-11 |
| Perforin | dG9 | Alexa 647 | Mouse IgG2b | 1:20 | Biolegend | 308110 |
| CD25 | K231.3B2 | — | Mouse IgG1 | 1:100 | Bio-Rad | MCA1736 |
| SLA-DR | 2E9/13 | — | Mouse IgG2b | 1:500 | Bio-Rad | MCA2314 |
| CD5 | b53b7 | — | Mouse IgG1 | 1:100 | in-house | — |
| MHC II | MSA3 | — | Mouse IgG2a | 1:100 | in-house | — |
| CD27 | b30c7 | — | Mouse IgG1 | 1:5 | in-house | — |
| gdTCR | PPT16 | — | Mouse IgG2b | 1:100 | in-house | — |
| CD8β | PG164A | — | Mouse IgG2a | 1:1000 | in-house | — |
| CD172a | 74-22-15 | — | Mouse IgG1 | 1:100 | in-house | — |
| α-IgG1 | RMG1-1 | BV421 | Rat IgG | 1:400 | Biolegend | 406616 |
| α-IgG2a | polyclonal | APC-Cy7 | Goat IgG | 1:250 | Southern Biotech | 1080-19 |
| α-IgG2b | polyclonal | PE-Cy7 | Goat IgG | 1:400 | Southern Biotech | 1090-17 |
